# Development and validation of the CARE-DM model to predict the cardiovascular risk in older persons with type 2 diabetes

**DOI:** 10.1093/eurjpc/zwaf296

**Published:** 2025-05-14

**Authors:** Valerie Aponte Ribero, Orestis Efthimiou, Heba Alwan, Douglas C Bauer, Séverine Henrard, Gérard Waeber, Pedro Marques-Vidal, Nicolas Rodondi, Cinzia Del Giovane, Baris Gencer

**Affiliations:** Institute of Primary Health Care (BIHAM), University of Bern, Mittelstrasse 43, Bern 3012, Switzerland; Graduate School for Health Sciences, University of Bern, Mittelstrasse 43, Bern 3012, Switzerland; Institute of Primary Health Care (BIHAM), University of Bern, Mittelstrasse 43, Bern 3012, Switzerland; Institute of Primary Health Care (BIHAM), University of Bern, Mittelstrasse 43, Bern 3012, Switzerland; Departments of Medicine and Epidemiology & Biostatistics, University of California San Francisco, San Francisco, CA, USA; Clinical Pharmacy & Pharmacoepidemiology Research Group, Louvain Drug Research Institute (LDRI), Université catholique de Louvain, Brussels 1200, Belgium; Institute of Health and Society (IRSS), Université catholique de Louvain, Brussels 1200, Belgium; Department of Medicine, Internal Medicine, Lausanne University Hospital (CHUV) and University of Lausanne, Lausanne 1011, Switzerland; Department of Medicine, Internal Medicine, Lausanne University Hospital (CHUV) and University of Lausanne, Lausanne 1011, Switzerland; Institute of Primary Health Care (BIHAM), University of Bern, Mittelstrasse 43, Bern 3012, Switzerland; Department of General Internal Medicine, Inselspital, Bern University Hospital, University of Bern, Bern 3010, Switzerland; Institute of Primary Health Care (BIHAM), University of Bern, Mittelstrasse 43, Bern 3012, Switzerland; Department of Medical and Surgical Sciences for Children and Adults, University-Hospital of Modena and Reggio Emilia, Modena 41124, Italy; Institute of Primary Health Care (BIHAM), University of Bern, Mittelstrasse 43, Bern 3012, Switzerland; Cardiology Division, Geneva University Hospitals, Rue Gabrielle-Perret-Gentil 6, 1205 Geneva, Switzerland; Department of Cardiology, Lausanne University Hospital (CHUV) and University of Lausanne, Rue du Bugnon 46, Lausanne 1011, Switzerland

**Keywords:** Cardiovascular diseases, Diabetes mellitus, Primary prevention, Heart disease risk factors

## Abstract

**Aims:**

No cardiovascular risk prediction model dedicated to individuals aged ≥70 years with diabetes is currently recommended by the European Society of Cardiology. We aimed to develop a new model, CArdiovascular Risk Estimation—Diabetes Mellitus (CARE-DM), to predict the risk of cardiovascular disease (CVD) in older adults with type 2 diabetes.

**Methods and results:**

We developed a model to predict the risk of incident CVD in participants aged ≥65 years with diabetes using data from four population-based prospective cohorts, accounting for the competing risk of non-cardiovascular death. Pre-specified predictors were age, gender, smoking status, alcohol consumption, body mass index, total and HDL cholesterol, use of antihypertensive, cholesterol-lowering and glucose-lowering medication, diabetes duration, and glycated haemoglobin. We assessed model performance using measures of calibration and discrimination. We used a 10-fold cross-validation and a bootstrapping approach to correct estimates for optimism and conducted an internal–external cross-validation. A total of 6943 participants (median age 72 years, 56% women) with diabetes were included in the model development. Over a median follow-up of 6.3 (interquartile range 3.7, 7.2) years, 1204 (17.3%) participants experienced a CVD event. Internal validation with optimism correction showed adequate model performance with a C-index of 0.65 (95% confidence interval 0.63–0.67), an observed-to-expected ratio of 1.01 (0.95–1.08), and a calibration slope of 1.13 (0.95–1.31) at 5 years.

**Conclusion:**

The new CARE-DM model allows prediction of the incident CVD risk in older adults with type 2 diabetes. Independent external validation should be conducted to confirm the model’s performance before implementation in clinical practice.

## Graphical Abstract

**Graphical Abstract:**
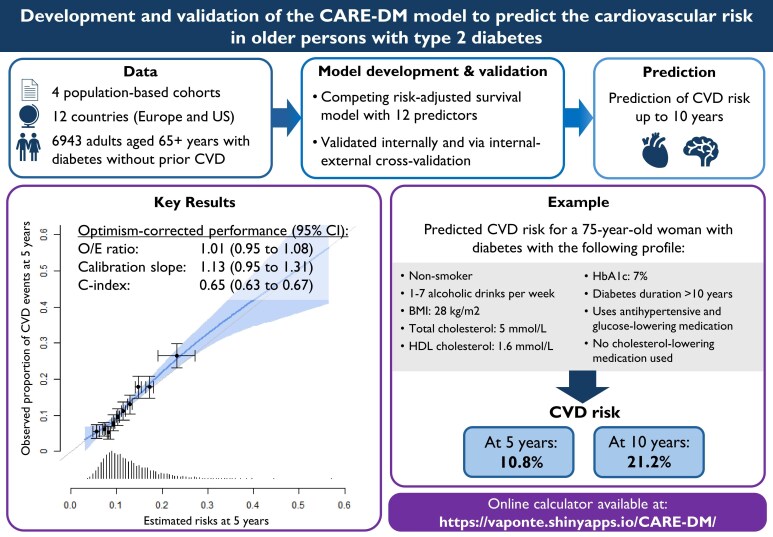

## Introduction

Cardiovascular disease (CVD) is a major of cause of morbidity and mortality in individuals with type 2 diabetes,^1^ especially in older adults.^2^ Cardiovascular prevention guidelines tailor treatment recommendations to the patient’s expected CVD risk,^3,4^ which is typically obtained via the use of established prognostic models.^5–7^ Risk stratification is particularly relevant in older age given the higher risk of CVD events, but also the higher likelihood of multimorbidity, polypharmacy, and treatment side effects.^8^ Properly developed risk scores that take into account the competing risk of non-cardiovascular death may help clinicians identify those who are at lower or higher risk in the older population. According to clinical guidelines, this information should be taken into consideration when making treatment decisions, in combination with other considerations such as life expectancy, frailty, polypharmacy, and patient preferences.^9^ However, so far, CVD prediction models developed for persons with diabetes have not focused on older adults or did not adjust for the competing risk of non-cardiovascular death,^10^ resulting in lower predictive performance in older adults.^11^

Although the European Society of Cardiology (ESC) recently introduced the SCORE2-OP model to predict the risk of CVD in adults aged ≥70 years in primary prevention, this model is not recommended for older adults with type 2 diabetes.^6^ Instead, the ESC recommends SCORE2-Diabetes for patients with diabetes; however, this model is only valid for individuals aged 40–69 years old, as it was developed as an extension of the SCORE2 model.^4,7^ Notably, no equivalent model based on SCORE2-OP has been established for older adults aged 70 years or above with diabetes. DIAL2 is another prediction model for diabetes in individuals 30–85 years of age.^12^ While it was mainly developed for the prediction of lifetime CVD risk, 10-year CVD risk can also be estimated, although ESC guidelines do not specifically recommend it for this purpose. In the USA, the PREVENT models are recommended for CVD risk prediction in the general population aged 30–79 years.^3,5^ However, unlike the ESC, no diabetes-specific model has been endorsed by the American Heart Association/American College of Cardiology guidelines.

Therefore, there is a need for a clinical CVD prediction tool for older adults with type 2 diabetes including diabetes-specific predictors such as haemoglobin A1c (HbA1c), diabetes duration, and diabetes medication use, using data from both the USA and Europe. We aimed to develop and validate a new model, CARE-DM (CArdiovascular Risk Estimation—Diabetes Mellitus), for use in primary prevention in adults aged ≥65 years with type 2 diabetes, using data from 12 countries provided by 4 population-based cohorts in Europe and the USA.

## Methods

The model was developed following recent guidance on clinical prediction model development^13^ and reported in accordance with the Transparent Reporting of a multivariable prediction model for Individual Prognosis Or Diagnosis (TRIPOD) guidelines.^14^

### Study design

The development of CARE-DM involved multiple steps. First, we derived model coefficients of predictors and baseline risks of the fatal and non-fatal CVD outcome using pooled individual participant data of four cohorts involving 6943 participants aged 65+ years with diabetes without prior CVD in 12 countries. Second, we assessed the model performance in internal validation and derived optimism-corrected performance estimates. Third, we further validated the model using an internal–external cross-validation procedure to gain insights on model performance in new settings. Fourth, we implemented an online tool to facilitate the calculation of CVD risk based on CARE-DM.

### Data sources

To develop the model, we used individual participant data from four prospective population-based cohort studies, namely, the Cohorte Lausannoise (CoLaus) study^15^; the Health, Aging, and Body Composition (Health ABC) study^16^ ; the Health and Retirement Study (HRS)^17^ ; and the Survey of Health, Ageing and Retirement in Europe (SHARE).^18,19^ These cohorts were selected based on their representativeness of older adults and data availability in terms of key predictors and outcomes. We have already used those data in a previous publication evaluating the risk of diabetes in older adults in an individual participant data meta-analysis.^2^

The CoLaus study was originally designed to evaluate the association between cardiovascular risk factors and CVD. It included 6734 participants from Lausanne (Switzerland).^15^ The study started in 2003 with follow-ups conducted every 5 years. HbA1c was first collected in the second follow-up, conducted between 2014 and 2018, which we used as baseline for the present study. From CoLaus, we included 236 participants for our analysis.

The Health ABC study was conducted in a cohort of 3075 older adults aged 70–79 years living in Memphis and Pittsburgh (USA), starting in 1997 with 16 years of follow-up, aimed at investigating risk factors for functional decline and changes in body composition.^16^ Since the coordinating centre indicated potential measurement errors of HbA1c at baseline, we used the Year 6 follow-up (conducted in 2002/3) as the baseline for our current study. Thus, from Health ABC, we included 386 participants at Year 6 for the present study.

The HRS study is a longitudinal panel study of approximately 20 000 older individuals living in the USA, designed to collect data on ageing in longitudinal surveys every 2 years starting from 1992.^17^ Biomarkers and information on cholesterol-lowering medication were first collected in the 2006 survey. For the present study, we defined baseline as the first survey from 2006 to 2019 when participants were aged 65+ years and participated in the collection of biomarkers. In total, 2860 participants were included from HRS.

The SHARE study is a longitudinal panel study on ageing, with over 140 000 older participants, conducted in 27 European countries and Israel.^18,19^ Starting in 2004, surveys were conducted every 2 years. For the present study, we used data from 11 countries (Belgium, Denmark, Estonia, France, Germany, Greece, Israel, Italy, Slovenia, Spain, and Switzerland), which collected biomarkers during the sixth survey (conducted in 2015)^20^; this was considered as baseline in our analyses. From SHARE, we included 3461 participants.

Details on specific data sets used from HRS and SHARE are available in Supplementary material online, *Table S1*. All included studies were approved by an ethical committee and obtained informed consent from its participants. An approval was obtained for the current study by the ethics committee in Bern (Switzerland, ID 2022-01263). The protocol is available on medRxiv.^21^

### Participants

We included participants aged 65 years and older with type 2 diabetes and without established CVD in model development. Participants were considered to have type 2 diabetes if they (i) had a diabetes diagnosis, (ii) used diabetes medication, or (iii) met any of the following diagnostic criteria: HbA1c > 6.5%, fasting glucose ≥ 7 mmol/L or oral glucose tolerance test measurement ≥ 11 mmol/L.^22^ The definition of diabetes in each of the included cohorts is presented in Supplementary material online, *Table S2*. Established CVD was defined as history of coronary heart disease, stroke, or peripheral arterial disease (history of peripheral arterial disease was only available in CoLaus and Health ABC). Definitions of diabetes and history of CVD were consistent with those applied in SCORE2-Diabetes.^7^ A flow diagram of participant selection for each study is presented in Supplementary material online, *Figure S1*.

### Outcome

Our aim was to predict the occurrence of incident fatal or non-fatal cardiovascular event over time. In all cohorts, the outcome was defined as the composite of cardiovascular death, non-fatal myocardial infarction, or non-fatal stroke.^4^ Details on outcome definition in each of the studies are provided in Supplementary material online, *Table S3*. For the development of the model, from each participant, we analysed the time from baseline until the first cardiovascular event, non-cardiovascular death, or study end. Non-cardiovascular deaths were considered as competing events in our analyses. Outcomes ascertainment is described for each study in Supplementary material online, *Table S3*.

### Predictors

Predictors of the model were pre-specified in the protocol and selected based on a review of commonly included predictors in published CVD prediction models, with a focus on those developed in individuals with diabetes, and of other potentially relevant variables that could improve model performance.^21^ We also considered the availability or ease of measurement in everyday clinical practice in our predictor selection as well as their availability in our cohorts. We included the following predictors in our model: age (continuous), gender (women vs. men), total and HDL cholesterol (continuous), smoking status (current vs. other), weekly alcohol consumption (<1 drink per week, 1–7 drinks per week, >7 drinks per week; categorized because continuous data was unavailable from Health ABC), body mass index (BMI, continuous), use of antihypertensive medication (yes vs. no), use of cholesterol-lowering (yes vs. no), use of glucose-lowering medication (yes vs. no), diabetes duration [≤5 years, 5–10 years, >10 years; categorized because duration had to be approximated for CoLaus (follow-ups were conducted in 5-year intervals)], and HbA1c (continuous). Details on predictor definitions and assessments are presented in Supplementary material online, *Table S4*. All predictors were pre-specified in the protocol,^21^ except for the use of cholesterol-lowering drugs, which was nevertheless added before starting any analyses and reviewing results. The decision to include this predictor was because of the high proportion of participants using cholesterol-lowering drugs for primary CVD prevention (50%, *Table 1*) and potential additional non-lipid lowering pleiotropic effects of statins. Although not a traditional CVD risk factor, we included alcohol consumption as a predictor in our model due to recommendations from ESC guidelines to limit alcohol intake, supported by evidence linking higher intake with increased cardiovascular morbidity and mortality as well as with an elevated risk of both hypo- and hyperglycaemia in individuals with diabetes.^9,23^ We did not include other candidate predictors such as race, history of atrial fibrillation, albumin-to-creatinine ratio, triglycerides, or systolic blood pressure, as they were not available in all cohorts. Renal function expressed in estimated glomerular filtration rate (eGFR) was included as a predictor in sensitivity analysis due to its inclusion in the SCORE2-Diabetes and PREVENT models.^5,7^

**Table 1:** Study and baseline characteristics of participants included in the CARE-DM model development

	Overall	CoLaus	Health ABC	HRS	SHARE
Countries		Switzerland	USA	USA	Belgium, Denmark, Estonia, France, Germany, Greece, Israel, Italy, Slovenia, Spain, Switzerland
Baseline years		2014–18	2002–03	2006–19	2015
*N*	6943	236	386	2860	3461
Follow-up time (years)	6.3 [3.7, 7.2]	3.8 [3.5, 4.0]	9.8 [6.0, 11.6]	6.3 [3.7, 10.2]	6.3 [3.2, 6.8]
Predictors at baseline					
Age (years)	72 [67, 78]	73 [70, 77]	78 [76, 80]	69 [66, 75]	73 [69, 79]
Women	3869 (56%)	114 (48%)	212 (55%)	1667 (58%)	1876 (54%)
Current smoker	486 (11%)	31 (15%)	16 (5%)	262 (17%)	177 (8%)
Number of alcoholic drinks per week					
<1 per week	4078 (64%)	69 (35%)	301 (78%)	1720 (73%)	1988 (58%)
1–7 per week	1639 (26%)	68 (35%)	62 (16%)	480 (20%)	1029 (30%)
>7 per week	665 (10%)	58 (30%)	22 (6%)	146 (6%)	439 (13%)
BMI (kg/m^2^)	28.8 [26.0, 32.3]	28.3 [25.4, 31.9]	30.7 [27.1, 34.9]	27.6 [25.1, 31.1]	28.8 [25.6, 32.8]
Use of antihypertensive medication	4608 (67%)	156 (66%)	276 (73%)	2071 (73%)	2105 (61%)
Use of cholesterol-lowering medication	3370 (50%)	112 (47%)	111 (30%)	1689 (62%)	1458 (42%)
Total cholesterol (mmol/L)	5.3 [4.6, 5.8]	5.0 [4.2, 5.6]	4.8 [4.2, 5.4]	4.7 [4.1, 5.5]	5.6 [5.2, 6.0]
HDL cholesterol (mmol/L)	1.5 [1.2, 1.7]	1.4 [1.1, 1.6]	1.4 [1.1, 1.7]	1.3 [1.0, 1.6]	1.7 [1.5, 1.8]
Diabetes duration					
≤5 years	1693 (33%)	54 (23%)	131 (44%)	784 (34%)	724 (32%)
5–10 years	1011 (20%)	84 (36%)	58 (19%)	518 (22%)	351 (16%)
>10 years	2383 (47%)	98 (42%)	109 (37%)	1007 (44%)	1169 (52%)
HbA1c (%)	6.6 [6.1, 7.0]	6.3 [5.8, 6.8]	6.5 [5.9, 7.1]	6.6 [6.0, 7.3]	6.6 [6.2, 6.9]
Use of diabetes medication	4789 (73%)	127 (54%)	190 (51%)	2092 (84%)	2380 (69%)
Outcomes during follow-up					
Cardiovascular event	1204 (17.3%)	20 (8.5%)	129 (33.4%)	579 (20.2%)	476 (13.8%)
Cardiovascular death^a^	605 (8.7%)	10 (4.2%)	81 (21.0%)	259 (9.1%)	255 (7.4%)
Non-fatal MI^a^	648 (9.3%)	6 (2.5%)	43 (11.1%)	384 (13.4%)	215 (6.2%)
Non-fatal stroke^a^	567 (8.2%)	6 (2.5%)	46 (11.9%)	316 (11.0%)	199 (5.7%)
Non-cardiovascular death	1524 (22.0%)	35 (14.8%)	156 (40.4%)	869 (30.4%)	464 (13.4%)

Numbers are presented as *n* (%) or median [Q1-Q3], calculated based on non-missing data.

BMI, body mass index; HbA1c, haemoglobin A1c; HDL, high-density lipoprotein; MI, myocardial infarction.

^a^Do not sum to total cardiovascular events as participants may have had more than one type of event.

### Statistical analysis

#### Sample size calculation

We calculated the required sample size to develop the prediction model in the protocol.^21^ We assumed a 5-year outcome prevalence of 15%, an *R*^2^ of 15%, and inclusion of a maximum of 19 predictors. Based on these assumptions, the minimum sample size required for the model was 1043 participants. A lower *R*^2^ of 10% would require 1614 participants. Thus, our sample size of 6943 participants was sufficient.

#### Model development

We performed multiple imputation by chained equation to impute missing predictor data across all studies for a total of 10 imputed data sets. The imputation model for missing predictors included the outcome indicator and Nelson–Aalen estimate of the cumulative hazard, as recommended.^24^ We developed the prediction model in each complete data set in a one-stage approach (i.e. individual participant data from all cohorts were pooled).^25^ We fitted a flexible parametric survival model (Royston–Parmar model) in which the log cumulative hazard was modelled with a natural cubic spline function of log time with two internal knots^26^ and compared it with a Weibull model. All predictors were included when comparing the models. We selected the Weibull model as the final model as it was simpler with equal predictive performance and had a lower Akaike information criterion (AIC) compared with the flexible parametric survival model. We accounted for the competing risk of non-CVD death by modelling the cause-specific cumulative incidence function using time-dependent weights (a parametric equivalent of the Fine–Gray model).^26^ The model included a single common intercept rather than a random intercept to account for clustering since there were too few studies to robustly estimate the random effect and baseline hazards were similar between studies.^27^ Continuous predictors were included with linear terms in the model since there was no strong evidence for nonlinearity based on visual checks and the AIC. For the final model, we combined the coefficients of the 10 imputed data sets using Rubin’s rules. To visualize the model predictions and the observed cumulative incidence functions, we classified participants into low (<2.5%), moderate (2.5–<5%), high (5–<10%), and very high (≥10%) 5-year risk of CVD. These categories were approximated as half of the 10-year risk thresholds defined by ESC guidelines for patients with diabetes.^4^ We developed an online calculator using the Shiny R package to facilitate use of the prediction model in clinical practice (available at https://vaponte.shinyapps.io/CARE-DM/). We present confidence intervals around the risk estimates in the online tool, following recent recommendations.^28^

#### Evaluation of the model performance

We assessed model performance at 5 and 10 years in terms of discrimination and calibration. Discrimination was assessed using Wolbers’ C-index, which accounts for competing risks (unlike Harrell’s C-index).^29^ Calibration was assessed with observed-to-expected ratio and calibration plots, intercept, and slope using the pseudo-observation approach to account for competing risks.^30^ Apparent model performance (both discrimination and calibration) was evaluated overall, by study and by subgroups defined by gender (women vs. men) and age (65–74 vs. ≥75 years). Clinical utility of the model was assessed via decision curve analysis.^31^

#### Model validation

The model was validated internally via bootstrapping with 500 samples to estimate optimism.^13^ Optimism-corrected performance statistics were calculated by subtracting the average optimism from apparent model performance. Due to long model run times, bootstrapping was performed only on a single imputation data set. We additionally performed 10-fold cross-validation on all imputation data sets.^13^ Moreover, we performed an internal–external cross-validation, in which we developed the model in three studies and tested it on the fourth, iteratively for each study (except for CoLaus as most participants were censored by 5 years; Supplementary material online, *Figure S2*). This approach has been recommended by recent prediction modelling guidelines over holding out a small number of studies from model development for external validation, as it allows for both full data utilization and assessment of model generalizability across different settings.^13,32^ Results from each iteration of the internal–external cross-validation were meta-analysed and heterogeneity was quantified via *I*^2^ and the *Q* test.

#### Sensitivity analyses

Sensitivity analyses were conducted to assess if model performance could be improved by including eGFR as a predictor. Additionally, since internal–external validation indicated potential heterogeneity in calibration across studies, we developed a model that included an indicator for low-, moderate-, or high-risk region. This model was developed by assigning each of the countries included in our cohorts to the risk region defined as in the SCORE2-Diabetes model (presented in Supplementary material online, *Table S5*) and including the indicator for risk region as a predictor in the model.^6,7,33^ We also provisionally compared the performance between CARE-DM, SCORE2-Diabetes, DIAL2, and PREVENT ASCVD models using 10-fold cross-validation. Since SCORE2-Diabetes and PREVENT ASCVD only provided 10-year risks, performance could not be compared for our primary 5-year outcome.

All analyses were conducted using R statistical software version 4.4.0.^34^ The statistical code is available at https://github.com/v-aponte-ribero/CARE-DM.

## Results

A total of 6943 older adults with type 2 diabetes and without established CVD were included in model development (*Table 1*). The median age of participants was 72 years [interquartile range (IQR) 67–78] and 56% were women. Over a median follow-up of 6.3 (IQR 3.7–7.2, range 0–15.2) years, 1204 (17.3%) participants had a fatal or non-fatal cardiovascular event and 1524 (22.0%) died of non-cardiovascular causes. The proportion of missing data ranged from 0 to 47% (for HDL cholesterol) (see Supplementary material online, *Table S6*). Baseline characteristics after imputation are presented in Supplementary material online, *Table S6*.

**Figure 1:**
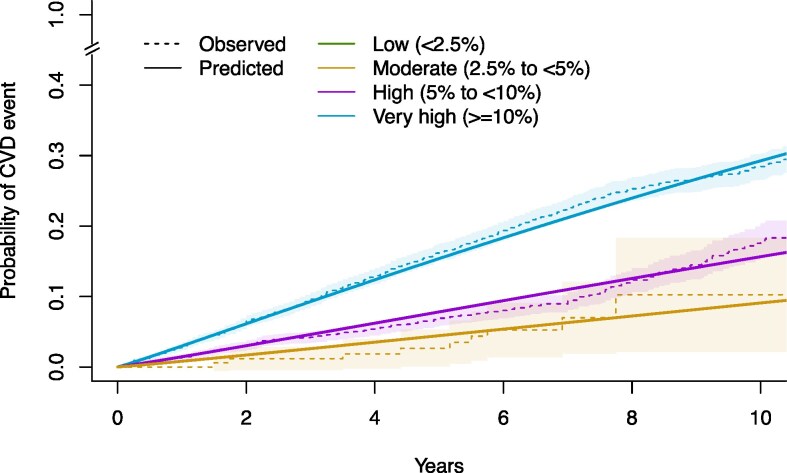
Observed vs. predicted cardiovascular disease risks by risk group. Risk groups were defined according to predicted 5-year risks based on the risk groups defined as in the European Society of Cardiology guidelines for patients with diabetes (10-year risks were divided in half to approximate 5-year risks).^4^ There were no participants in the low-risk group, as no participants had a 5-year cardiovascular disease risk of <2.5% in our data set. Observed and estimated risks were derived from the first imputation data set.

Coefficients and sub-distribution hazard ratios (i.e. exponentiated coefficients) of each predictor in the final model are presented in *Table 2*. The variance–covariance matrix of model coefficients is available in Supplementary material online, *Table S7*. Based on the model predictions of 5-year risk of CVD and our classification system, 0% of participants were classified at low risk (5-year risk < 2.5%), 3% at moderate risk (5-year risk between 2.5 and <5%), 39% at high risk (5-year risk between 5 and <10%), and 58% at very high risk (5-year risk > 10%) (*Figure 1*).

**Table 2:** Coefficients and hazard ratios of CARE-DM (Weibull model, *N* = 6943 participants)

		β-Coefficients (95% CI)	Sub-distribution hazard ratios (95% CI)
Age (per year)		0.054 (0.046–0.063)	1.06 (1.05–1.07)
Women (vs. men)		−0.337 (−0.461 to −0.214)	0.71 (0.63–0.81)
Current smoker (vs. no current smoker)		0.318 (0.135–0.501)	1.37 (1.14–1.65)
Number of alcoholic drinks per week	<1 per week	Reference	Reference
	1–7 per week	−0.246 (−0.393 to −0.100)	0.78 (0.68–0.90)
	>7 per week	−0.116 (−0.327–0.095)	0.89 (0.72–1.10)
BMI (per 1 kg/m^2^)		0.019 (0.009–0.029)	1.02 (1.01–1.03)
Use of antihypertensive medication (vs. no use)		0.268 (0.136–0.399)	1.31 (1.15–1.49)
Use of cholesterol-lowering medication (vs. no use)		−0.123 (−0.245 to −0.002)	0.88 (0.78–1.00)
Total cholesterol (per 1 mmol/L)		0.006 (−0.061–0.074)	1.01 (0.94–1.08)
HDL cholesterol (per 1 mmol/L)		−0.066 (−0.236–0.103)	0.94 (0.79–1.11)
Diabetes duration	<5 years	Reference	Reference
	5–10 years	0.038 (−0.140–0.215)	1.04 (0.87–1.24)
	>10 years	0.221 (0.074–0.369)	1.25 (1.08–1.45)
HbA1c (per 1%-point)		0.080 (0.034–0.125)	1.08 (1.03–1.13)
Use of diabetes medication (vs. no use)		−0.039 (−0.193–0.114)	0.96 (0.82–1.12)
Weibull parameter 1 (gamma0)		−6.651 (−6.959 to −6.344)	—
Weibull parameter 2 (gamma1)		1.065 (1.011–1.118)	—

Weibull parameter as defined in the flexsurvspline function from the flexsurv package.^35^ Weibull parameter 1 (gamma0) corresponds to -shape*log(scale), and Weibull parameter 2 (gamma1) corresponds to shape in the ‘dweibull’ function in base R.

BMI, body mass index; HbA1c, haemoglobin A1c; HDL, high-density lipoprotein.

**Figure 2:**
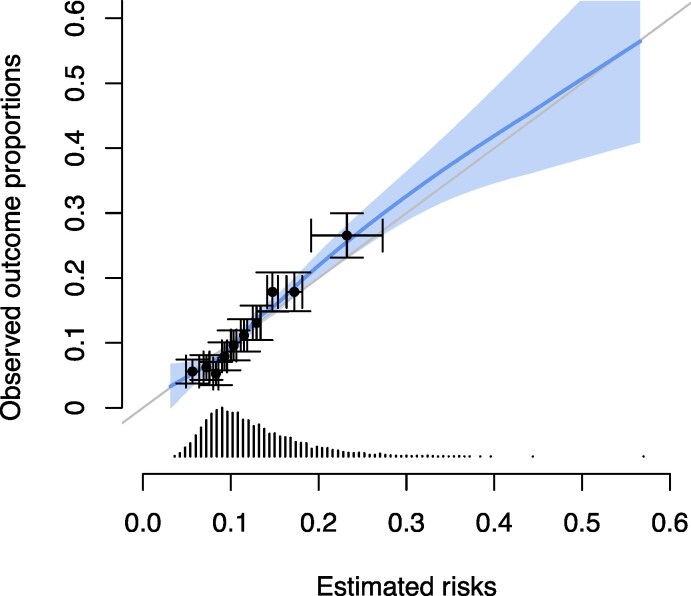
Calibration plot (apparent performance). The calibration plot displays the estimated risk of cardiovascular disease vs. the observed proportion with a cardiovascular disease event at 5 years. The bold line shows a smoothed calibration curve, derived based on pseudo-values which account for the competing risks of non-cardiovascular death.^30^ The dots display deciles of the predicted risks vs. observed probabilities. Horizontal error bars indicate the standard deviation of predicted risks in each decile. Vertical error bars indicate the standard error of the observed probabilities.

*Table 3* shows the 5-year apparent and optimism-corrected model performance. In general, internal validation did not show strong evidence for optimism in performance. The optimism-corrected C-index was 0.65 [95% confidence interval (CI) 0.63–0.67] in the bootstrap validation and 0.65 (0.59–0.70) in the 10-fold cross-validation. In terms of calibration, optimism-corrected observed-to-expected ratio was 1.01 (0.95–1.08) in the bootstrap validation and 1.01 (0.82–1.25) in the 10-fold cross-validation. The calibration slope was estimated at 1.13 (0.95–1.31) and 1.14 (0.59–1.69), respectively. *Figure 2* displays the calibration plot for the 5-year risk of CVD. Apparent calibration at 5 years was similar when evaluated in each study separately (see Supplementary material online, *Table S8*). However, there was some evidence of heterogeneity in the C-index (*I*^2^ = 63%; *Q*-test *P* = 0.046) with higher C-index in European (SHARE, 0.67, 95% CI 0.64–0.67) compared with US cohorts (Health ABC, 0.62, 95% CI 0.55–0.68; HRS, 0.63, 95% CI 0.60–0.66). Apparent performance was similar across subgroups of age (<75 vs. ≥75 years) and between men and women (see Supplementary material online, *Table S9*).

**Table 3:** Apparent, optimism-corrected, and 10-fold cross-validation model performance at 5 years

	Apparent performance (95% CI)	Optimism (95% CI)^a^	Optimism-corrected performance (95% CI)^a^	10-fold cross-validation performance (95% CI)
Observed-to-expected ratio	1.01 (0.95–1.08)	0.00 (−0.07–0.07)	1.01 (0.95–1.08)	1.01 (0.82–1.25)
Calibration intercept	0.05 (−0.03–0.12)	0.01 (−0.07–0.08)	0.04 (−0.04–0.11)	0.03 (−0.21–0.27)
Calibration slope	1.18 (1.00–1.36)	0.05 (−0.12–0.22)	1.13 (0.95–1.31)	1.14 (0.59–1.69)
C-index	0.65 (0.63–0.67)	0.01 (−0.01–0.03)	0.65 (0.63–0.67)	0.65 (0.59–0.70)

The ideal value for the observed-to-expected ratio is 1 (perfect calibration-in-the-large) with values < 1 indicating that predicted risks are overestimated and values > 1 indicating that predicted risks are underestimated. Similarly, for the calibration intercept, 0 is the ideal value with values < 0 indicating overestimation of risks and values > 0 indicating underestimation of risks. For calibration slope, the ideal value is 1, with values < 1 indicating overfitting (predicted risks are overestimated for individuals at high risk and underestimated for individuals at low risk) and values > 1 indicating underfitting (predicted risks are underestimated for individuals at high risk and overestimated for individuals at low risk). The C-index ranges from 0 to 1 with a value of 0.5 corresponding to random chance and 1 to perfect discrimination.

^a^Optimism was estimated using bootstrap validation on the first imputation data set only due to long model run time. Optimism-corrected performance was calculated by subtracting the mean optimism from the apparent performance.

Internal–external cross-validation showed that models were stable with similar hazard ratios for the predictors estimated in each of the validation iterations (see Supplementary material online, *Table S10*). However, there was evidence of heterogeneity in the observed-to-expected ratio (*I*^2^ = 73%; *Q*-test *P* = 0.02; Supplementary material online, *Figure S4*), indicating that the model’s performance on calibration-in-the-large varies across population. There was no strong evidence for heterogeneity in the calibration intercept, slope, or C-index (see Supplementary material online, *Figure S3*). Calibration plots for each iteration of the internal–external cross-validation are provided in Supplementary material online, *Figure S4*. The decision curve analysis indicated that use of the CARE-DM model would be clinically useful at threshold probabilities defined by clinical guidelines (the ESC defines very high risk as a 10-year risk of 20%, corresponding to approximately a 10% risk at 5-years) (see Supplementary material online, *Figure S5*).^4^

At 10 years, observed-to-expected ratio was 1.03 (0.85–1.24), calibration slope was 0.72 (0.25–1.18), and C-index was 0.61 (0.55–0.66) in optimism-corrected results from 10-fold cross-validation (see Supplementary material online, *Table S11*). Corresponding results in the same data were 1.05 (0.87–1.27), 0.59 (0.20–0.98), and 0.59 (0.53–0.65) for SCORE2-Diabetes; 1.13 (0.93–1.36), 0.79 (0.26–1.30) and 0.61 (0.56–0.67) for DIAL2; and 0.63 (0.52–0.76), −0.36 (0.74–0.02), and 0.55 (0.49–0.61) for PREVENT ASCVD (see Supplementary material online, *Table S12*). In participants aged ≥75 years, the C-index was 0.59 (0.50–0.66) for CARE-DM, 0.54 (0.46–0.62) for SCORE2-Diabetes, 0.58 (0.49–0.65) for DIAL2, and 0.53 (0.45–0.61) for PREVENT ASCVD.

Sensitivity analysis of a model that included eGFR as a predictor did not improve model performance (see Supplementary material online, *Table S13*). Coefficients for the model that included an additional indicator for low-, moderate-, or high-risk region, defined as in SCORE2-Diabetes,^7^ are presented in Supplementary material online, *Table S14*. The variance–covariance matrix is available in Supplementary material online, *Table S15*. Apparent performance of this model was similar to the main model (see Supplementary material online, *Table S16*).

We provide an interactive online calculator for CARE-DM, available at https://vaponte.shinyapps.io/CARE-DM/. Instructions for manual calculation of the CVD risk using CARE-DM are available in Supplementary material online, *Appendix S2*.

## Discussion

We developed the new CARE-DM model to support CVD risk stratification of older adults, i.e. aged 65 years and older, with type 2 diabetes in primary cardiovascular prevention. Our tool predicts the risk of CVD for up to 10 years using routinely collected predictors. We validated this tool via internal and internal–external cross-validation and developed an online calculator to facilitate its use.

The CARE-DM model fills a gap in clinical guidelines with respect to older adults with type 2 diabetes, as current CVD risk prediction models recommended by European and US guidelines are either not applicable for persons over 69 years of age (SCORE2-Diabetes) or were not specifically developed for persons with diabetes (PREVENT).^5,7^ The discriminative performance of CARE-DM (C-index 0.65, 95% CI 0.63–0.67) is comparable to other CVD prediction models in older adults, such as SCORE2-OP (0.66, 95% CI 0.65–0.66).^6^ Importantly, our decision curve analysis supports the clinical utility of CARE-DM at a relevant risk thresholds of 10% 5-year risk, which corresponds to the ‘very high’ risk category for which guidelines recommend lower cholesterol targets and treatment intensification.^7^

Mukamal *et al*.^36^ also developed a model to estimate the risk of CVD in older adults with diabetes from the USA with a similar discriminative performance (C-index of 0.65). However, in contrast to our model, competing risks were not taken into account during development and performance assessment. Not accounting for competing risks during model development may result in overestimation of CVD risk, i.e. older adults might be predicted to be at higher risk than they really are, leading to potentially unnecessary treatment.^30^ Moreover, our model was developed on a larger sample size (6943 vs. 782 older adults), using data from 12 countries. In contrast to SCORE2-Diabetes and the model by Mukamal *et al*.,^7,36^ and similar to the PREVENT equations,^5^ we included use of antihypertensive and cholesterol-lowering medications as predictors, which is relevant for older populations due to polypharmacy and associated adverse effects, and potential pleiotropic effects of medications such as statins.^8^ The number of predictors included in CARE-DM (12 predictors) is comparable with SCORE2-Diabetes (9 predictors) and the ASCVD PREVENT equations (11 in the basic model; 14 in the enhanced model).^5,7^

Our model provides predictions of absolute CVD risk, helping physicians in stratifying older patients into CVD risk categories to inform decisions on risk factor treatment.^9^ US and European cardiovascular prevention guidelines recommend that physicians should consider absolute CVD risk for patients with diabetes when setting lipoprotein cholesterol and blood pressure targets, as well as when making decisions on prescribing of statins, PCSK9 inhibitors, antihypertensives, or glucose-lowering treatment such as GLP-1 receptor agonists or SGLT-2 inhibitors.^4^ In older adults, where the risk of non-cardiovascular death is high, these treatment decisions should also take into account the individual’s life expectancy, comorbidities, frailty, and patient preferences.^9^ Since CARE-DM is implemented as an online tool, it can further facilitate shared decision-making between patients and physicians.

We opted to focus on the 5-year risk of CVD rather than the 10-year risk, which is often used in other CVD prediction models, since only two of our studies had a follow-up of 10 years; life expectancy is typically shorter in this older age group, and studies have shown that health consumers and general practitioners have a preference for presentation of 5-year over 10-year CVD risks.^37^ Nevertheless, as predictions of 5-year risk cannot be computed using SCORE2-Diabetes and PREVENT ASCVD, we compared their performance with our new model CARE-DM on the prediction of 10-year risk. CARE-DM showed evidence of better performance compared with SCORE2-Diabetes in participants ≥ 75 years of age (due to small sample sizes in the 10-fold cross-validation, the power to detect differences between the models was very low). There was also evidence for a better performance compared with PREVENT ASCVD in the overall development data, while performance was similar to DIAL2. However, these results must be interpreted with caution, as a comparison of 5-year risk prediction was not possible, and although we used 10-fold cross-validation to get out-of-sample predictions for CARE-DM, the data used were still similar to the development data. A comparison of these models in an external data set is needed to firmly establish comparative performance.

In line with recent recommendations, we did not validate our model in an external data set in this study, but used all available data for development and conducted internal–external cross-validation.^13,32^ Results from the internal–external cross-validation indicated that our model may underestimate CVD risk in US settings similar to HRS. Therefore, we additionally presented a model that included an indicator for low-, moderate-, or high-risk region (see Supplementary material online, *Table S4*, regions defined as in SCORE-Diabetes).^7^ This model may show better calibration in future external validations, particularly in US data sets.

Our study has some limitations. We cannot exclude potential misclassification as data from the HRS and SHARE cohorts collected self-reported outcomes; this may have led to reduced model performance. Previous studies compared self-reported CVD event rates in HRS with other data sources with medical verification and found that rates were similar for stroke as well as for myocardial infarction when compared with acute coronary syndrome.^38,39^ We also did not find strong evidence for miscalibration in Health ABC (this study adjudicated outcomes) during internal–external cross-validation, and calibration-in-the-large was excellent when applying the SCORE2-Diabetes model in our data. Further, although of importance for individuals with diabetes, non-fatal heart failure or peripheral artery disease could not be included in the outcome due to data unavailability across cohorts. We also did not have data on some potentially relevant predictors (e.g. retinopathy, geriatric variables, specific diabetes drug classes) nor on the duration of risk factors other than diabetes, such as hypertension, which might have improved model performance. In order to classify participants by risk groups, we halved the thresholds for 10-year risks from the ESC guidelines, as no 5-year thresholds were given.^4^ We acknowledge that this is only an approximation and that 5-year thresholds may be higher than those assumed here. Moreover, the model was developed based on data from primarily low and moderate CVD risk regions in Europe and the USA and may therefore require recalibration in regions with very low or high levels of CVD. External validation should be conducted, ideally by an independent team, to confirm model performance in target populations. Although use of potent novel diabetes drugs such as SGLT-2 inhibitors and GLP-1 receptor agonists is still limited in older adults for primary cardiovascular prevention,^40^ model recalibration may be necessary in the future if a significant increase in their uptake is observed. Strengths of our study include the large sample size of older adults with diabetes due to pooling data from four studies; the inclusion of predictors that are routinely available in clinical practice; and the use of robust methodology accounting for competing risks in model development and performance assessment.

In conclusion, we developed and validated the CARE-DM model to predict the risk of CVD events at up to 10 years in older adults aged 65 years and older with type 2 diabetes. This model may help clinicians in stratifying patients according to their CVD risk and facilitate conversations on disease management for primary cardiovascular prevention between patients and physicians.

## Supplementary Material

zwaf296_Supplementary_Data

## Data Availability

The data underlying this article were provided by the data owners of each cohort under licence/by permission. Information on how researchers can access the data is listed as follows: *CoLaus*: Researchers affiliated to a public or private research institution who comply with the CoLaus|PsyCoLaus standards can submit a research application to research.colaus@chuv.ch or research.psycolaus@chuv.ch. Proposals will be evaluated by a Scientific Committee (SC). Detailed information for gaining data access is available at www.colaus-psycolaus.ch/professionals/how-to-collaborate/. *Health ABC*: To use Health ABC data, a proposal must be submitted to NIAHealthABCAdministration@mail.nih.gov. Detailed information for gaining data access is available at https://www.nia.nih.gov/healthabc-study. *HRS*: Data from this study are available for researchers after registration at https://hrs.isr.umich.edu/. *SHARE*: Data from this study are available for researchers after registration on https://share-eric.eu/data/.

## Previous presentations

An abstract of this work was accepted for moderated e-Poster presentation at the ESC Preventive Cardiology 2025 Congress and at the SGAIM Spring Congress 2025.
