# Supplementary material for: Development and validation of the CARE-DM model to predict the cardiovascular risk in older persons with type 2 diabetes
Source: Eur J Prev Cardiol. 2025 May 14;33(1):44–52. doi: 10.1093/eurjpc/zwaf296 (PMC12771341; doi:10.1093/eurjpc/zwaf296)
Supplement: zwaf296_Supplementary_Data [file zwaf296_supplementary_data.zip › CVD prediction model_Supplement1_EJPC_revision_clean.pdf]

SUPPLEMENTARY APPENDIX 1

Supplement to: “Development and validation of the CARE-DM model to predict the cardiovascular risk in older persons with type 2 diabetes”.

Contents

Supplementary Table 1 HRS and SHARE datasets used 3

Supplementary Table 2 Diabetes definition by cohort 3

Supplementary Table 3 Outcome definition and ascertainment by cohort 4

Supplementary Table 4 Predictor definitions and assessments 5

Supplementary Table 5 List of countries by risk regions 7

Supplementary Table 6 Missing data and imputed values of baseline characteristics 8

Supplementary Table 7 Variance-covariance matrix of model coefficients 10

Supplementary Table 8 Apparent performance at 5 years by study 11

Supplementary Table 9 Age and gender subgroup analyses of apparent performance at 5 years 11

Supplementary Table 10 Overview of models in each iteration of the internal-external cross-validation 12

Supplementary Table 11 Apparent model and 10-fold cross-validation performance of CARE-DM at 10-years 13

Supplementary Table 12 Comparison CARE-DM to SCORE2-Diabetes and PREVENT performance for prediction of 10-year risk using 10-fold cross-validation 13

Supplementary Table 13 Apparent and 10-fold cross-validation model performance at 5-years for model including eGFR 14

Supplementary Table 14 Coefficients and hazard ratios of CARE-DM including risk region indicator 15

Supplementary Table 15 Variance-covariance matrix of model coefficients for the model including a risk region indicator 16

Supplementary Table 16 Apparent model performance at 5-years for the model including a risk region indicator 17
Supplementary Figure 1 Participant flow chart 18
Supplementary Figure 2 Internal-external cross-validation approach 19
Supplementary Figure 3 Results from internal-external cross-validation........ 20
Supplementary Figure 4 Calibration plots from internal-external cross-validation........ 21
Supplementary Figure 5 Decision curve analysis 21
References........ 22

Supplementary Table 1 HRS and SHARE datasets used

Study	Datasets used
HRS	Cross-wave tracker file;[1]<br>2006-2020 Core Datasets;[2-9]<br>2006-2020 Exit Datasets;[10-17]<br>2006-2016 Biomarker Dataset;[18-23]<br>2016 Venous Blood Study Dataset[24]
SHARE	Wave 6-9 Dataset;[25-30]<br>Wave 6 Dried Blood Spots Dataset[31]

Supplementary Table 2 Diabetes definition by cohort

	**CoLaus**	**Health ABC**	**HRS**	**SHARE**
**Type 2 diabetes definition**	Defined as any of the following[32]:<br>- Self-reported physician diagnosis of diabetes ("Have you ever been told by a doctor that you have diabetes?")<br>- Diabetes medication use (oral medication or insulin)<br>- HbA1c ≥6.5%<br>- Fasting plasma glucose ≥7.0 mmol/L	Defined as any of the following:<br>- Self-reported physician diagnosis of diabetes ("Has a doctor ever told you that you have any of the following conditions…?" Diabetes or sugar diabetes)<br>- Diabetes medication use (oral medication or insulin)<br>- HbA1c ≥6.5%<br>- Fasting plasma glucose ≥126 mg/dL<br>- Oral glucose tolerance test ≥11 mmol/l	Defined as any of the following:<br>- Self-reported physician diagnosis of diabetes ("Has a doctor ever told you that you have diabetes or high blood sugar?")<br>- Diabetes medication use (oral medication or insulin)<br>- HbA1c ≥6.5%	Defined as any of the following:<br>- Self-reported physician diagnosis of diabetes ("Has a doctor ever told you that you had any of the conditions on this card? Diabetes or high blood sugar")<br>- Diabetes medication use (oral medication or insulin)<br>- HbA1c ≥6.5%

Supplementary Table 3 Outcome definition and ascertainment by cohort

Study	Outcome definition	Outcome ascertainment
CoLaus	A composite of:<br>- Cardiovascular death (deaths from cerebrovascular origin; cardiac death including fatal myocardial infarction and cardiac sudden deaths; vascular deaths including aortic dissection, valvular heart disease, fatal arrhythmia and cardiac failure)<br>- Nonfatal myocardial infarction (STEMI or NSTEMI)<br>- Nonfatal stroke (I61 - nontraumatic intracerebral hemorrhage and I63 - cerebral infarction)[33]	Cardiovascular events were ascertained from participants during follow-up visits/calls and medical records were obtained. Deaths were ascertained from population registers or reports from a relative. Information on cause of death was taken from general practitioners, hospital charts, death certificates, autopsy records or verbal autopsy from relatives (if other sources were unavailable). Events were adjudicated by local adjudication committee.
Health ABC	A composite of:<br>- Cardiovascular death (fatal myocardial infarction or CHD; cerebrovascular origin; atherosclerotic disease, other than coronary or cerebrovascular; other cardiovascular disease such as valvular heart disease)<br>- Nonfatal myocardial infarction (hospitalisation for myocardial infarction)<br>- Nonfatal stroke (hospitalisation for stroke)	Cardiovascular events were ascertained from participants during regular follow-up visits/calls, medical records and obituaries. Events were adjudicated until August 2012 by a committee of clinicians by reviewing hospital records, death certificates and other data. Possible or definite cardiovascular events were included in the current study.
HRS	A composite of:<br>- Cardiovascular death (death due to heart problems and death due to stroke)<br>- Nonfatal myocardial infarction (self-report: "In the last two years, have you had a heart attack or myocardial infarction")<br>- Nonfatal stroke (self-report: "In the last two years, have you had a stroke")	Cardiovascular events were ascertained from participants or proxies during regular surveys by asking whether the participant had a 'heart attack or myocardial infarction' or a 'stroke' since their last interview as well as the date of the most recent event. Deaths and cause of deaths were ascertained via 'exit' interviews from relatives or other informants and linkages to the National Death Index.
SHARE	A composite of:<br>- Cardiovascular death (death due a heart attack, a stroke or other cardiovascular related illness)<br>- Nonfatal myocardial infarction (self-report: "Have you had a heart attack since we last interviewed you")<br>- Nonfatal stroke (self-report: "Have you had a stroke or been diagnosed with cerebral vascular disease since we last interviewed you")	Cardiovascular events were ascertained from participants or proxies during regular surveys by asking whether the participant had a 'heart attack' or a 'stroke' since their last interview as well as the date of the most recent event. Deaths and cause of deaths were ascertained via 'end-of-life' interviews from relatives or other informants.

Supplementary Table 4 Predictor definitions and assessments

**Predictor**	**Definition and assessments.**
Age	Continuous age at baseline in years.
Gender	Men versus women; captured as gender (Health ABC, HRS, SHARE) or sex (CoLaus).
Alcohol consumption	Number of alcoholic drinks per week, categorized into <1 drink per week, 1 to 7 drinks per week and >1 drink per day, as no continuous variable was available in Health ABC.<br>Alcohol consumption was captured as follows in each study:<br>CoLaus: Units of alcoholic drinks in the past seven days (continuous)<br>Health ABC: Number of drinks in a typical week during the past 12 months (categorical: None; An occasional drink, but less than once a week; 1-3 per week; 4-7 per week; 8-14 per week; 15-21 per week; 22-27 per week; 28 or more per week). As this variable was not assessed at Year 6, the value at screening was used.<br>HRS: Number of days per week in which participant had any alcohol to drink and number of drinks on the days when the participant drinks, in the last three month (continuous).<br>SHARE: Number of units of alcoholic drinks in the last 7 days (continuous).
BMI	Calculated as weight in kilograms divided by the square of height in meters.
Use of antihypertensive medication	Current use vs no current use of antihypertensive medication.<br>Use of antihypertensive medication was captured as follows in each study:<br>CoLaus: Antihypertensive drug treatment and drugs with ATC codes C02, C03, C07, C08, C09.<br>Health ABC: Any antihypertensive agent, including beta-adrenergic blockers, alpha-adrenergic blockers, hypotensive agents, ACE inhibitors, angiotensin II hypotensive agents, hydralazine, calcium channel blockers, and diuretics.<br>HRS: Question to participants: "In order to lower your blood pressure, are you now taking any medication?".<br>SHARE: Question to participants: "Do you currently take drugs at least once a week for problems mentioned on this card? Drugs for high blood pressure".
Use of cholesterol-lowering medication	Current use vs no current use of cholesterol-lowering medication.<br>Use of cholesterol-lowering medication was captured as follows in each study:<br>CoLaus: Hypolipidemic drug treatment and drugs with ATC codes C10<br>Health ABC: Any antilipemic medication including statins, high dose niacin and ezetimibe.<br>HRS: Question to participants: "Do you regularly take prescription medications for any of the following common health problems: To help lower your cholesterol?".<br>SHARE: Question to participants: "Do you currently take drugs at least once a week for problems mentioned on this card? Drugs for high blood cholesterol".
Total and HDL cholesterol	Total and HDL cholesterol were expressed in mmol/L and were measured as follows in each study:<br>CoLaus: Measured from fresh venous blood sample.<br>Health ABC: Measured from venous blood samples. Cholesterol values in mg/dL were converted to mmol/L.<br>HRS: Measured from dried blood spots. Cholesterol values in mg/dL were converted to mmol/L. NHANES equivalent values were used for the analyses.<br>SHARE: Measured from dried blood spots. Values corrected for fieldwork conditions, spot size, and storage time, then converted to standard equivalents, were used for the analyses.
Diabetes duration	Diabetes duration in years, categorized by ≤ 5 years, 5 to 10 years, and > 10 years, as CoLaus did not collect data on diabetes duration at later follow-ups.

Predictor	Definition and assessments.
	Diabetes duration was captured as follows in each study:<br>CoLaus: For participants with diabetes at initiation of CoLaus, age at diabetes diagnosis was collected and subtracted from baseline age to derive diabetes duration. For participants who had diabetes at the 1st or 2nd follow-up, diabetes duration was approximated by assessing whether the participant had diabetes in previous follow-ups which were conducted approximately every 5 years.<br>Health ABC: Participant-reported age at diabetes diagnosis was collected and subtracted from baseline age to derive diabetes duration.<br>HRS: The participant-reported year at which diabetes was first diagnosed was collected and subtracted from the baseline year to derive diabetes duration.<br>SHARE: Participant-reported age at diabetes diagnosis was collected and subtracted from baseline age to derive diabetes duration.
Use of diabetes medication	Current use vs no current use of diabetes medication (including insulin and oral medications).<br>Use of diabetes medication was captured as follows in each study:<br>CoLaus: Antidiabetic drug treatment and drugs with ATC code A10.<br>Health ABC: Any diabetes medication including insulins and antidiabetic agents.<br>HRS: Question to participants: "In order to treat or control your [diabetes/blood sugar], are you now taking medication that you swallow?" and "Are you now using insulin shots or a pump?".<br>SHARE: Question to participants: "Do you currently take drugs at least once a week for problems mentioned on this card? Drugs for diabetes".
HbA1c	HbA1c were expressed in % and were measured as follows in each study:<br>CoLaus: Measured from fresh venous blood sample by high performance liquid chromatography using a Bio-Rad, D-10TM system.<br>Health ABC: Measured from venous blood samples using the Bio-Rad Variant analyzer.<br>HRS: Measured from dried blood spots using various systems, depending on the year of data collection. NHANES equivalent values were used for the analyses.<br>SHARE: Measured from dried blood spots by high performance liquid chromatography (HPLC) systems. Values corrected for fieldwork conditions, spot size, and storage time were used for the analyses.

Supplementary Table 5 List of countries by risk regions

Risk region	Countries
Low risk	○ **Belgium**<br>○ **Denmark**<br>○ **France**<br>○ **Israel**<br>○ Luxembourg<br>○ Netherlands<br>○ Norway<br>○ **Spain**<br>○ **Switzerland**<br>○ United Kingdom of Great Britain and Northern Ireland
Moderate risk	○ Austria<br>○ Cyprus<br>○ Finland<br>○ **Germany**<br>○ **Greece**<br>○ Iceland<br>○ Ireland<br>○ **Italy**<br>○ Malta<br>○ Portugal<br>○ San Marino<br>○ **Slovenia**<br>○ Sweden<br>○ **United States of America**
High risk	○ Albania<br>○ Bosnia and Herzegovina<br>○ Croatia<br>○ Czechia<br>○ **Estonia**<br>○ Hungary<br>○ Kazakhstan<br>○ Poland<br>○ Slovakia<br>○ Turkey

Risk regions defined as in the SCORE2-Diabetes and SCORE2-OP models.[33,34] Countries in bold were included in model development.

Supplementary Table 6 Missing data and imputed values of baseline characteristics

		Overall	CoLaus	Health ABC	HRS	SHARE
**Age**						
	N (%) missing	0	0	0	0	0
**Gender**						
	N (%) missing	0	0	0	0	0
**Current smoker**						
	N (%) missing	2’578 (37.1%)	25 (10.6%)	54 (14.0%)	1’285 (44.9%)	1214 (35.1%)
	% after imputation	12%	14%	5%	15%	9%
**Number of alcoholic drinks per week**						
	N (%) missing	561 (8.1%)	41 (17.4%)	1 (0.3%)	514 (18.0%)	5 (0.1%)
% after imputation	< 1 per week	64%	38%	78%	72%	58%
	1 to 7 per week	26%	34%	16%	21%	30%
	> 1 per day	10%	28%	6%	7%	13%
**BMI**						
	N (%) missing	559 (8.1%)	20 (8.5%)	51 (13.2%)	335 (11.7%)	153 (4.4%)
	Median (Q1, Q3) after imputation	28.8 (25.6, 32.9)	28.8 (25.8, 32.2)	28.2 (25.4, 31.9)	30.8 (27.2, 34.9)	27.6 (25.0, 31.1)
**Use of antihypertensive medication**						
	N (%) missing	39 (0.6%)	0	10 (2.6%)	28 (1.0%)	1 (0.0%)
	% after imputation	67%	66%	74%	73%	61%
**Use of cholesterol-lowering medication**						
	N (%) missing	167 (2.4%)	0	10 (2.6%)	155 (5.4%)	2 (0.1%)
	% after imputation	49%	47%	30%	61%	42%
**Total cholesterol (mmol/L)**						
	N (%) missing	2’184 (31.5%)	18 (7.6%)	71 (18.4%)	840 (29.4%)	1’255 (36.3%)
	Median (Q1, Q3) after imputation	5.2 (4.5, 5.8)	5.0 (4.3, 5.6)	4.9 (4.3, 5.5)	4.8 (4.1, 5.5)	5.5 (5.0, 6.0)

		Overall	CoLaus	Health ABC	HRS	SHARE
**HDL cholesterol (mmol/L)**						
N (%) missing		3’263 (47.0%)	18 (7.6%)	72 (18.7%)	1’015 (35.5%)	2’158 (62.4%)
Median (Q1, Q3) after imputation		1.5 (1.2, 1.7)	1.4 (1.2, 1.7)	1.4 (1.1, 1.7)	1.3 (1.1, 1.6)	1.6 (1.4, 1.8)
**Diabetes duration**						
N (%) missing		1’856 (26.7%)	0	88 (22.8%)	551 (19.3%)	1’217 (35.2%)
% after imputation	≤ 5 years	33%	23%	49%	35%	30%
	5 to 10 years	20%	36%	18%	22%	17%
	> 10 years	47%	42%	33%	43%	52%
**Use of diabetes medication**						
N (%) missing		386 (5.6%)	0	10 (2.6%)	370 (12.9%)	6 (0.2%)
% after imputation		73%	54%	51%	83%	69%
**HbA1c (%)**						
N (%) missing		2’470 (35.6%)	18 (7.6%)	80 (20.7%)	863 (30.2%)	1’509 (43.6%)
Median (Q1, Q3) after imputation		6.6 (6.1, 7.1)	6.3 (5.8, 6.8)	6.5 (6.0, 7.2)	6.6 (6.1, 7.3)	6.6 (6.2, 7.0)

Abbreviations: BMI, body mass index; HbA1c, hemoglobin A1c; HDL, high-density lipoprotein

Supplementary Table 7 Variance-covariance matrix of model coefficients

	gamma0	gamma1	Age	Gender	BMI	1-7 alcoholic drinks per week	>7 alcoholic drinks per week	Smoker	Antihypertensive drugs	Cholesterol-lowering drugs	Diabetes duration 5-10 years	Diabetes duration >10 years	Diabetes drugs	HbA1c	Total cholesterol	HDL cholesterol
gamma0	0.025183	-0.003460	-0.000118	-0.001961	0.000020	-0.002598	-0.002722	-0.002009	-0.002626	-0.001017	-0.003255	-0.002592	-0.001870	-0.000221	-0.000368	-0.001686
gamma1	-0.003460	0.000748	-0.000001	-0.000027	-0.000001	0.000073	0.000045	0.000008	-0.000006	0.000002	0.000149	0.000136	-0.000065	0.000008	0.000011	0.000196
Age	-0.000118	-0.000001	0.000020	-0.000004	0.000007	0.000012	0.000051	0.000136	0.000004	0.000016	-0.000037	-0.000040	0.000011	0.000013	0.000009	-0.000035
Gender	-0.001961	-0.000027	-0.000004	0.004282	-0.000047	0.001154	0.002348	-0.000011	-0.000444	-0.000307	0.000353	0.000674	-0.000208	0.000002	-0.000349	-0.001769
BMI	0.000020	-0.000001	0.000007	-0.000047	0.000029	0.000001	-0.000004	0.000120	-0.000035	-0.000011	0.000019	0.000015	-0.000051	0.000007	0.000015	0.000076
1-7 alcoholic drinks per week	-0.002598	0.000073	0.000012	0.001154	0.000001	0.006301	0.001976	-0.000702	0.000055	-0.000189	0.000953	0.000841	-0.000464	0.000103	-0.000323	0.000264
>7 alcoholic drinks per week	-0.002722	0.000045	0.000051	0.002348	-0.000004	0.001976	0.012376	-0.000274	-0.000166	-0.000539	-0.000171	0.000385	0.000125	0.000161	-0.000372	-0.002451
Smoker	-0.002009	0.000008	0.000136	-0.000011	0.000120	-0.000702	-0.000274	0.013671	0.000543	0.000295	-0.000119	0.000319	-0.001012	0.000279	0.000724	-0.001741
Antihypertensive drugs	-0.002626	-0.000006	0.000004	-0.000444	-0.000035	0.000055	-0.000166	0.000543	0.004519	-0.000631	0.000034	-0.000010	-0.000321	0.000113	0.000141	0.000242
Cholesterol-lowering drugs	-0.001017	0.000002	0.000016	-0.000307	-0.000011	-0.000189	-0.000539	0.000295	-0.000631	0.004082	-0.000073	-0.000115	-0.000497	-0.000068	0.000686	0.000206
Diabetes duration 5-10 years	-0.003255	0.000149	-0.000037	0.000353	0.000019	0.000953	-0.000171	-0.000119	0.000034	-0.000073	0.011902	0.005543	-0.003715	-0.000006	-0.000193	0.001903
Diabetes duration >10 years	-0.002592	0.000136	-0.000040	0.000674	0.000015	0.000841	0.000385	0.000319	-0.000010	-0.000115	0.005543	0.007376	-0.003915	-0.000076	-0.000100	-0.000712
Diabetes drugs	-0.001870	-0.000065	0.000011	-0.000208	-0.000051	-0.000464	0.000125	-0.001012	-0.000321	-0.000497	-0.003715	-0.003915	0.007466	-0.000337	0.000405	0.000068
HbA1c	-0.000221	0.000008	0.000013	0.000002	0.000007	0.000103	0.000161	0.000279	0.000113	-0.000068	-0.000006	-0.000076	-0.000337	0.000739	-0.000258	0.000423
Total cholesterol	-0.000368	0.000011	0.000009	-0.000349	0.000015	-0.000323	-0.000372	0.000724	0.000141	0.000686	-0.000193	-0.000100	0.000405	-0.000258	0.001680	-0.001805
HDL cholesterol	-0.001686	0.000196	-0.000035	-0.001769	0.000076	0.000264	-0.002451	-0.001741	0.000242	0.000206	0.001903	-0.000712	0.000068	0.000423	-0.001805	0.013725

Supplementary Table 8 Apparent performance at 5 years by study

	Observed-to-expected ratio (95% CI)	Calibration intercept (95% CI)	Calibration slope performance (95% CI)	C-index (95% CI)
**HRS**	1.06 (0.95 to 1.17)	0.08 (-0.04 to 0.20)	1.22 (0.93 to 1.51)	0.63 (0.60 to 0.66)
**CoLaus***	-	-	-	-
**Health ABC**	1.02 (0.81 to 1.29)	0.06 (-0.20 to 0.32)	1.57 (0.59 to 2.55)	0.62 (0.55 to 0.68)
**SHARE**	0.98 (0.89 to 1.08)	0.02 (-0.09 to 0.13)	1.14 (0.90 to 1.39)	0.67 (0.64 to 0.70)
**Meta-analysed (RE model)**	1.01 (0.95 to 1.08)	0.05 (-0.03 to 0.13)	1.19 (1.00 to 1.37)	0.65 (0.60 to 0.67)
**Heterogeneity: I^2; τ^2; p-value**	0%; 0.000; 0.748	0%; 0.000; 0.753	0%; 0.000; 0.684	63%; 0.013; 0.046

The ideal value for the observed-to-expected ratio is 1 (perfect calibration-in-the-large) with values <1 indicating that predicted risks are overestimated and values >1 indicating that predicted risks are underestimated. Similarly, for the calibration intercept, 0 is the ideal value with values <0 indicating overestimation of risks and values >1 indicating underestimation of risks. For calibration slope, the ideal value is 1, with values <1 indicating overfitting (predicted risks are overestimated for individuals at high risk and underestimated for individuals at low risk) and values >1 indicating underfitting (predicted risks are underestimated for individuals at high risk and overestimated for individuals at low risk). The C-index ranges from 0 to 1 with a value of 0.5 corresponding to random chance and 1 to perfect discrimination. *Performance measures not estimated for CoLaus due to only 6 participants still at risk by 5-years.

Abbreviations: RE, random effects

Supplementary Table 9 Age and gender subgroup analyses of apparent performance at 5 years

Subgroup	Observed-to-expected ratio (95% CI)	Calibration intercept (95% CI)	Calibration slope performance (95% CI)	C-index (95% CI)
**Age**				
**65 to 74 years**	0.95 (0.86 to 1.06)	-0.03 (-0.14 to 0.08)	1.17 (0.80 to 1.54)	0.61 (0.58 to 0.64)
**75+ years**	1.05 (0.96 to 1.15)	0.08 (-0.02 to 0.18)	1.16 (0.87 to 1.44)	0.63 (0.60 to 0.65)
**Gender**				
**Men**	1.04 (0.95 to 1.14)	0.06 (-0.05 to 0.16)	1.08 (0.83 to 1.33)	0.64 (0.61 to 0.66)
**Women**	0.99 (0.90 to 1.09)	0.04 (-0.07 to 0.14)	1.32 (1.04 to 1.6)	0.65 (0.63 to 0.68)

Ideal values are 1 for observed-to-expected ratio and calibration slope, and 0 for calibration intercept. The C-index ranges from 0 to 1 with a value of 0.5 corresponding to random chance and 1 to perfect discrimination.

Supplementary Table 10 Overview of models in each iteration of the internal-external cross-validation

		Final model	IECV 1	IECV 2	IECV 3
**Development datasets**		All (HRS, CoLaus, Health ABC, SHARE)	CoLaus, Health ABC, SHARE	HRS, CoLaus, SHARE	HRS, CoLaus, Health ABC
**Validation dataset**		-	HRS (moderate-risk region)	Health ABC (moderate-risk region)	SHARE (low-, moderate- and high-risk regions)
**Number of patients in validation dataset**		-	2'860	386	3'461
**Number of CVD events in validation dataset**		-	579 (20%)	129 (33%)	476 (14%)
**Predictors**		**Sub-distribution hazard ratios (95% CI)**			
**Age (per year)**		1.06 (1.05 to 1.07)	1.07 (1.06 to 1.08)	1.05 (1.05 to 1.06)	1.05 (1.04 to 1.06)
**Women (vs. men)**		0.71 (0.63 to 0.81)	0.60 (0.51 to 0.72)	0.72 (0.63 to 0.82)	0.79 (0.67 to 0.93)
**Current smoker (vs. no current smoker)**		1.37 (1.14 to 1.65)	1.37 (1.03 to 1.83)	1.41 (1.17 to 1.70)	1.28 (1.02 to 1.60)
**Number of alcoholic drinks per week**	**< 1 per week**	Reference	Reference	Reference	Reference
	**1 to 7 per week**	0.78 (0.68 to 0.90)	0.79 (0.65 to 0.97)	0.78 (0.67 to 0.91)	0.76 (0.62 to 0.94)
	**> 7 per week**	0.89 (0.72 to 1.10)	0.95 (0.73 to 1.23)	0.88 (0.71 to 1.10)	0.77 (0.56 to 1.06)
**BMI (per 1 kg/m^2)**		1.02 (1.01 to 1.03)	1.02 (1.00 to 1.04)	1.02 (1.01 to 1.03)	1.01 (1.00 to 1.03)
**Use of antihypertensive medication (vs. no use)**		1.31 (1.15 to 1.49)	1.39 (1.16 to 1.66)	1.31 (1.14 to 1.51)	1.19 (1.00 to 1.41)
**Use of cholesterol-lowering medication (vs. no use)**		0.88 (0.78 to 1.00)	0.92 (0.77 to 1.09)	0.91 (0.8 to 1.04)	0.81 (0.7 to 0.95)
**Total cholesterol (per 1 mmol/L)**		1.01 (0.94 to 1.08)	1.05 (0.95 to 1.17)	1.00 (0.93 to 1.07)	1.00 (0.93 to 1.09)
**HDL cholesterol (per 1 mmol/L)**		0.94 (0.79 to 1.11)	0.94 (0.73 to 1.20)	0.97 (0.81 to 1.16)	0.95 (0.77 to 1.17)
**Diabetes duration**	**< 5 years**	Reference	Reference	Reference	Reference
	**5 to 10 years**	1.04 (0.87 to 1.24)	1.11 (0.85 to 1.46)	1.06 (0.87 to 1.28)	0.99 (0.80 to 1.23)
	**> 10 years**	1.25 (1.08 to 1.45)	1.27 (1.01 to 1.60)	1.27 (1.09 to 1.49)	1.24 (1.04 to 1.48)
**HbA1c (per 1%-point)**		1.08 (1.03 to 1.13)	1.07 (1.00 to 1.15)	1.09 (1.04 to 1.14)	1.08 (1.02 to 1.14)
**Use of diabetes medication (vs. no use)**		0.96 (0.82 to 1.12)	0.89 (0.71 to 1.10)	0.98 (0.83 to 1.16)	0.97 (0.80 to 1.18)

Abbrevations: BMI, body mass index; HbA1c, hemoglobin A1c; HDL, high-density lipoprotein; IECV, internal-external cross-validation

Supplementary Table 11 Apparent model and 10-fold cross-validation performance of CARE-DM at 10-years

	Apparent performance	10-fold cross-validation performance
**Observed-to-expected ratio (95% CI)**	1.03 (0.97 to 1.09)	1.03 (0.85 to 1.24)
**Calibration intercept (95% CI)**	0.00 (-0.07 to 0.07)	-0.01 (-0.24 to 0.22)
**Calibration slope (95% CI)**	0.76 (0.61 to 0.91)	0.72 (0.25 to 1.18)
**C-index (95% CI)**	0.61 (0.59 to 0.63)	0.61 (0.55 to 0.66)

Ideal values are 1 for observed-to-expected ratio and calibration slope, and 0 for calibration intercept. The C-index ranges from 0 to 1 with a value of 0.5 corresponding to random chance and 1 to perfect discrimination.

Supplementary Table 12 Comparison CARE-DM to SCORE2-Diabetes and PREVENT performance for prediction of 10-year risk using 10-fold cross-validation

	CARE-DM	SCORE2-Diabetes	DIAL2	PREVENT ASCVD*
**Overall population**				
Observed-to-expected ratio (95% CI)	1.03 (0.85 to 1.24)	1.05 (0.87 to 1.27)	1.13 (0.93 to 1.36)	0.63 (0.52 to 0.76)
Calibration intercept (95% CI)	-0.01 (-0.24 to 0.22)	0.00 (-0.23 to 0.24)	0.11 (-0.13 to 0.34)	-1.22 (-1.43 to -1.02)
Calibration slope (95% CI)	0.72 (0.25 to 1.18)	0.59 (0.20 to 0.98)	0.79 (0.29 to 1.30)	-0.36 (-0.74 to 0.02)
C-index (95% CI)	0.61 (0.55 to 0.66)	0.59 (0.53 to 0.65)	0.61 (0.56 to 0.67)	0.55 (0.49 to 0.61)
**Age ≥75 years**				
Observed-to-expected ratio (95% CI)	0.98 (0.77 to 1.23)	0.99 (0.79 to 1.26)	1.08 (0.86 to 1.37)	0.78 (0.62 to 0.98)
Calibration intercept (95% CI)	-0.06 (-0.35 to 0.23)	-0.06 (-0.36 to 0.23)	0.07 (-0.22 to 0.36)	-0.38 (-0.67 to -0.09)
Calibration slope (95% CI)	0.64 (-0.09 to 1.38)	0.45 (-0.15 to 1.06)	0.69 (-0.21 to 1.59)	0.07 (-0.55 to 0.68)
C-index (95% CI)	0.59 (0.50 to 0.66)	0.54 (0.46 to 0.62)	0.58 (0.49 to 0.65)	0.53 (0.45 to 0.61)

Performance measures of all models were compared in 10-fold cross-validation. Models could not be compared for the 5-year risk as outcomes at 5 years were not reported for SCORE2-Diabetes and PREVENT. Ideal values are 1 for observed-to-expected ratio and calibration slope, and 0 for calibration intercept. The C-index ranges from 0 to 1 with a value of 0.5 corresponding to random chance and 1 to perfect discrimination. *The simplified PREVENT ASCVD model was used, as the survival model could not be replicated with the published information.

Supplementary Table 13 Apparent and 10-fold cross-validation model performance at 5-years for model including eGFR

	**Apparent performance (95% CI)**	**10-fold cross-validation performance (95% CI)**
**Observed-to-expected ratio**	1.01 (0.95 to 1.08)	1.01 (0.82 to 1.25)
**Calibration intercept**	0.05 (-0.03 to 0.12)	0.03 (-0.21 to 0.27)
**Calibration slope**	1.17 (1.00 to 1.35)	1.13 (0.59 to 1.66)
**C-index**	0.66 (0.64 to 0.68)	0.65 (0.59 to 0.70)

Ideal values are 1 for observed-to-expected ratio and calibration slope, and 0 for calibration intercept. The C-index ranges from 0 to 1 with a value of 0.5 corresponding to random chance and 1 to perfect discrimination.

Supplementary Table 14 Coefficients and hazard ratios of CARE-DM including risk region indicator

		β-coefficients (95% CI)	Sub-distribution hazard ratios (95% CI)
**Risk region**	**Low**	Reference	Reference
	**Moderate**	0.234 (0.077 to 0.391)	1.264 (1.080 to 1.478)
	**High**	0.124 (-0.178 to 0.426)	1.132 (0.837 to 1.531)
**Age (per year)**		0.056 (0.047 to 0.065)	1.058 (1.048 to 1.067)
**Gender**	**Men**	Reference	Reference
	**Women**	-0.343 (-0.467 to -0.219)	0.710 (0.627 to 0.804)
**Current smoker**	**No**	Reference	Reference
	**Yes**	0.317 (0.134 to 0.500)	1.373 (1.143 to 1.649)
**Number of alcoholic drinks per week**	**< 1 per week**	Reference	Reference
	**1 to 7 per week**	-0.234 (-0.381 to -0.087)	0.791 (0.683 to 0.917)
	**> 7 per week**	-0.084 (-0.296 to 0.128)	0.919 (0.743 to 1.137)
**BMI (per 1 kg/m^2)**		0.018 (0.008 to 0.028)	1.018 (1.008 to 1.029)
**Use of antihypertensive medication**	**No**	Reference	Reference
	**Yes**	0.252 (0.121 to 0.384)	1.287 (1.128 to 1.468)
**Use of cholesterol-lowering medication**	**No**	Reference	Reference
	**Yes**	-0.112 (-0.234 to 0.011)	0.894 (0.791 to 1.011)
**Total cholesterol (per 1 mmol/L)**		0.014 (-0.054 to 0.081)	1.014 (0.948 to 1.085)
**HDL cholesterol (per 1 mmol/L)**		-0.027 (-0.198 to 0.144)	0.974 (0.821 to 1.155)
**Diabetes duration**	**< 5 years**	Reference	Reference
	**5 to 10 years**	0.054 (-0.124 to 0.231)	1.055 (0.884 to 1.260)
	**> 10 years**	0.247 (0.100 to 0.395)	1.281 (1.105 to 1.484)
**HbA1c (per 1%-point)**		0.075 (0.029 to 0.120)	1.078 (1.030 to 1.128)
**Use of diabetes medication**	**No**	Reference	Reference
	**Yes**	-0.049 (-0.202 to 0.104)	0.952 (0.817 to 1.110)
**Weibull parameter 1 (gamma0)**		-6.818 (-7.147 to -6.489)	-
**Weibull parameter 2 (gamma1)**		1.057 (1.003 to 1.110)	-

Abbreviations: BMI, body mass index; HbA1c, hemoglobin A1c; HDL, high-density lipoprotein

Weibull parameter as defined in the flexsurvspline function from the flexsurv package.[35] Weibull parameter 1 (gamma0) corresponds to -shape*log(scale) and Weibull parameter 2 (gamma1) corresponds to shape in the 'dweibull' function in base R.

Supplementary Table 15 Variance-covariance matrix of model coefficients for the model including a risk region indicator

	gamma0	gamma1	Age	Gender	BMI	1-7 alcoholic drinks per week	>7 alcoholic drinks per week	Smoker	Antihypertensive drugs	Cholesterol-lowering drugs	Diabetes duration 5-10 years	Diabetes duration >10 years	Diabetes drugs	HbA1c	Total cholesterol	HDL cholesterol
gamma0	0.029408	-0.003287	-0.000149	-0.001712	0.000029	-0.003109	-0.003380	-0.001960	-0.002282	-0.001455	-0.003992	-0.003393	-0.001535	-0.000115	-0.000539	-0.003075
gamma1	-0.003287	0.000745	-0.000002	-0.000022	0.000000	0.000053	0.000033	0.000044	0.000004	-0.000007	0.000097	0.000107	-0.000050	0.000015	0.000006	0.000112
Age	-0.000149	-0.000002	0.000020	-0.000005	0.000007	0.000015	0.000053	0.000135	0.000002	0.000020	-0.000026	-0.000032	0.000008	0.000013	0.000009	-0.000021
Gender	-0.001712	-0.000022	-0.000005	0.004307	-0.000046	0.001129	0.002350	-0.000008	-0.000428	-0.000328	0.000293	0.000616	-0.000182	0.000000	-0.000353	-0.001886
BMI	0.000029	0.000000	0.000007	-0.000046	0.000029	-0.000002	-0.000010	0.000124	-0.000034	-0.000013	0.000022	0.000017	-0.000053	0.000008	0.000015	0.000074
1-7 alcoholic drinks per week	-0.003109	0.000053	0.000015	0.001129	-0.000002	0.006399	0.002096	-0.000695	0.000022	-0.000132	0.001046	0.000975	-0.000548	0.000095	-0.000305	0.000447
>7 alcoholic drinks per week	-0.003380	0.000033	0.000053	0.002350	-0.000010	0.002096	0.012578	-0.000384	-0.000234	-0.000460	-0.000060	0.000501	0.000100	0.000133	-0.000393	-0.002241
Smoker	-0.001960	0.000044	0.000135	-0.000008	0.000124	-0.000695	-0.000384	0.013638	0.000537	0.000309	0.000031	0.000374	-0.001042	0.000274	0.000714	-0.001738
Antihypertensive drugs	-0.002282	0.000004	0.000002	-0.000428	-0.000034	0.000022	-0.000234	0.000537	0.004551	-0.000671	-0.000003	-0.000048	-0.000317	0.000122	0.000143	0.000118
Cholesterol-lowering drugs	-0.001455	-0.000007	0.000020	-0.000328	-0.000013	-0.000132	-0.000460	0.000309	-0.000671	0.004145	-0.000022	-0.000047	-0.000523	-0.000071	0.000691	0.000334
Diabetes duration 5-10 years	-0.003992	0.000097	-0.000026	0.000293	0.000022	0.001046	-0.000060	0.000031	-0.000003	-0.000022	0.011890	0.005675	-0.003773	-0.000004	-0.000101	0.002119
Diabetes duration >10 years	-0.003393	0.000107	-0.000032	0.000616	0.000017	0.000975	0.000501	0.000374	-0.000048	-0.000047	0.005675	0.007529	-0.003975	-0.000074	-0.000046	-0.000407
Diabetes drugs	-0.001535	-0.000050	0.000008	-0.000182	-0.000053	-0.000548	0.000100	-0.001042	-0.000317	-0.000523	-0.003773	-0.003975	0.007519	-0.000356	0.000386	-0.000141
HbA1c	-0.000115	0.000015	0.000013	0.000000	0.000008	0.000095	0.000133	0.000274	0.000122	-0.000071	-0.000004	-0.000074	-0.000356	0.000747	-0.000257	0.000386
Total cholesterol	-0.000539	0.000006	0.000009	-0.000353	0.000015	-0.000305	-0.000393	0.000714	0.000143	0.000691	-0.000101	-0.000046	0.000386	-0.000257	0.001669	-0.001670
HDL cholesterol	-0.003075	0.000112	-0.000021	-0.001886	0.000074	0.000447	-0.002241	-0.001738	0.000118	0.000334	0.002119	-0.000407	-0.000141	0.000386	-0.001670	0.014198

Supplementary Table 16 Apparent model performance at 5-years for the model including a risk region indicator

	Observed-to-expected ratio (95% CI)	Calibration intercept (95% CI)	Calibration slope (95% CI)	C-index (95% CI)
**Overall**	1.02 (0.95 to 1.09)	0.05 (-0.02 to 0.13)	1.17 (1.00 to 1.35)	0.65 (0.63 to 0.68)
**By study**				
**HRS**	1.02 (0.92 to 1.13)	0.04 (-0.08 to 0.16)	1.21 (0.92 to 1.49)	0.63 (0.60 to 0.66)
**CoLaus***	-	-	-	-
**Health ABC**	0.98 (0.77 to 1.24)	0.01 (-0.25 to 0.27)	1.61 (0.63 to 2.60)	0.62 (0.55 to 0.68)
**SHARE**	1.02 (0.92 to 1.12)	0.06 (-0.05 to 1.13)	1.13 (0.89 to 1.37)	0.67 (0.64 to 0.70)
**Meta-analysed (RE model)**	1.02 (0.95 to 1.09)	0.04 (-0.03 to 0.12)	1.17 (0.99 to 1.35)	0.64 (0.61 to 0.67)
**Heterogeneity: I^2; τ^2; p-value**	0%; 0; 0.988	0%; 0; 0.908	0%; 0; 0.450	54%; 0.010; 0.090

Ideal values are 1 for observed-to-expected ratio and calibration slope, and 0 for calibration intercept. The C-index ranges from 0 to 1 with a value of 0.5 corresponding to random chance and 1 to perfect discrimination. *Performance measures not estimated for CoLaus due to only 6 participants still at risk by 5-years.

Abbreviations: RE, random effects

Supplementary Figure 1 Participant flow chart

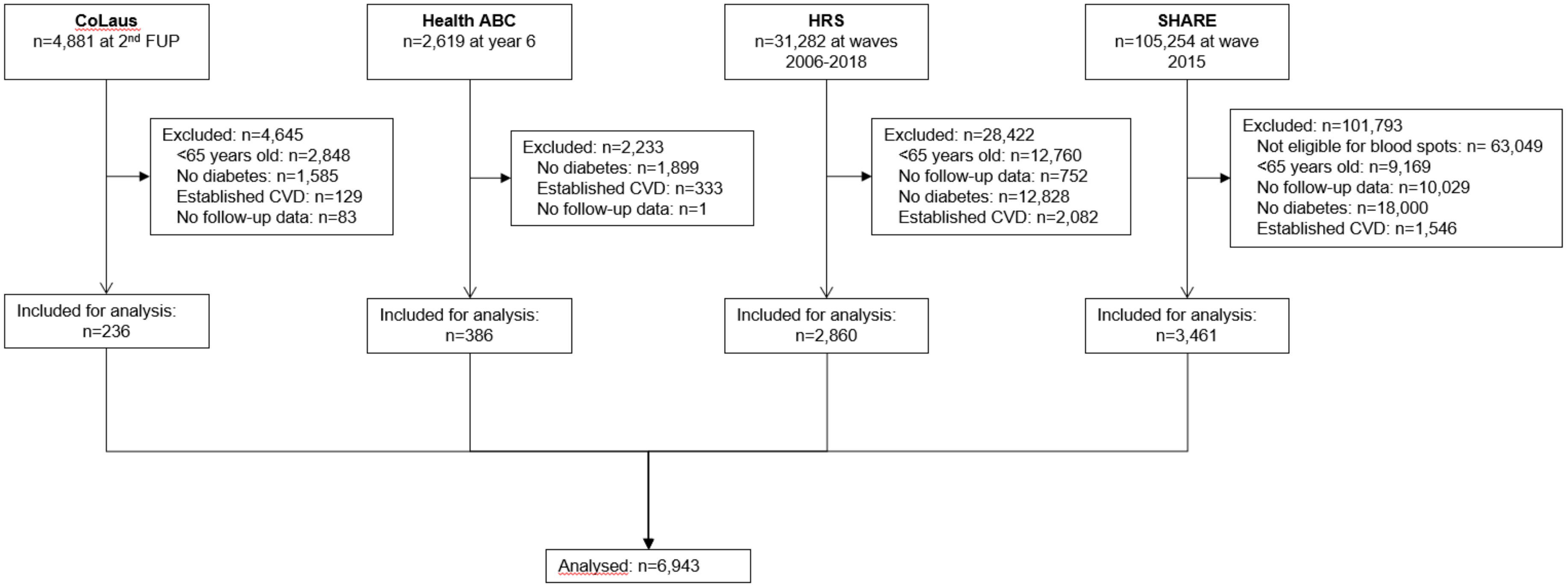

Abbreviations: CVD, cardiovascular disease, FUP, follow-up

Supplementary Figure 2 Internal-external cross-validation approach

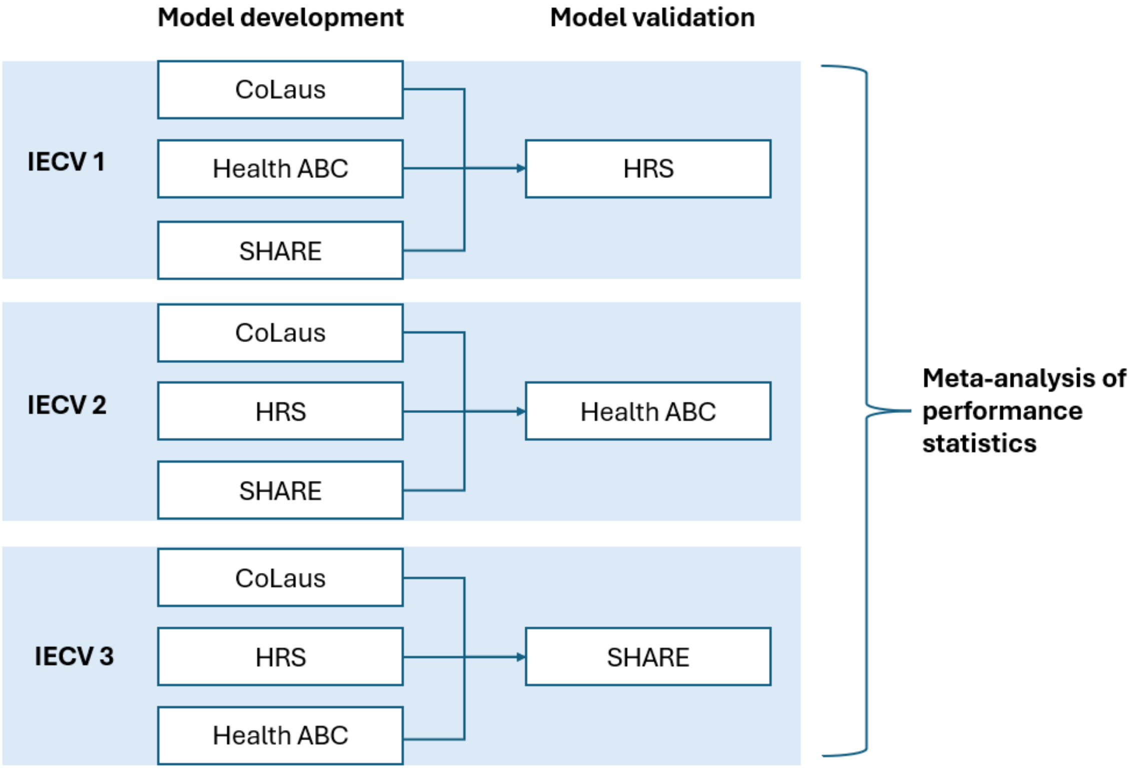

Abbreviations: IECV, internal-external cross-validation

Supplementary Figure 3 Results from internal-external cross-validation

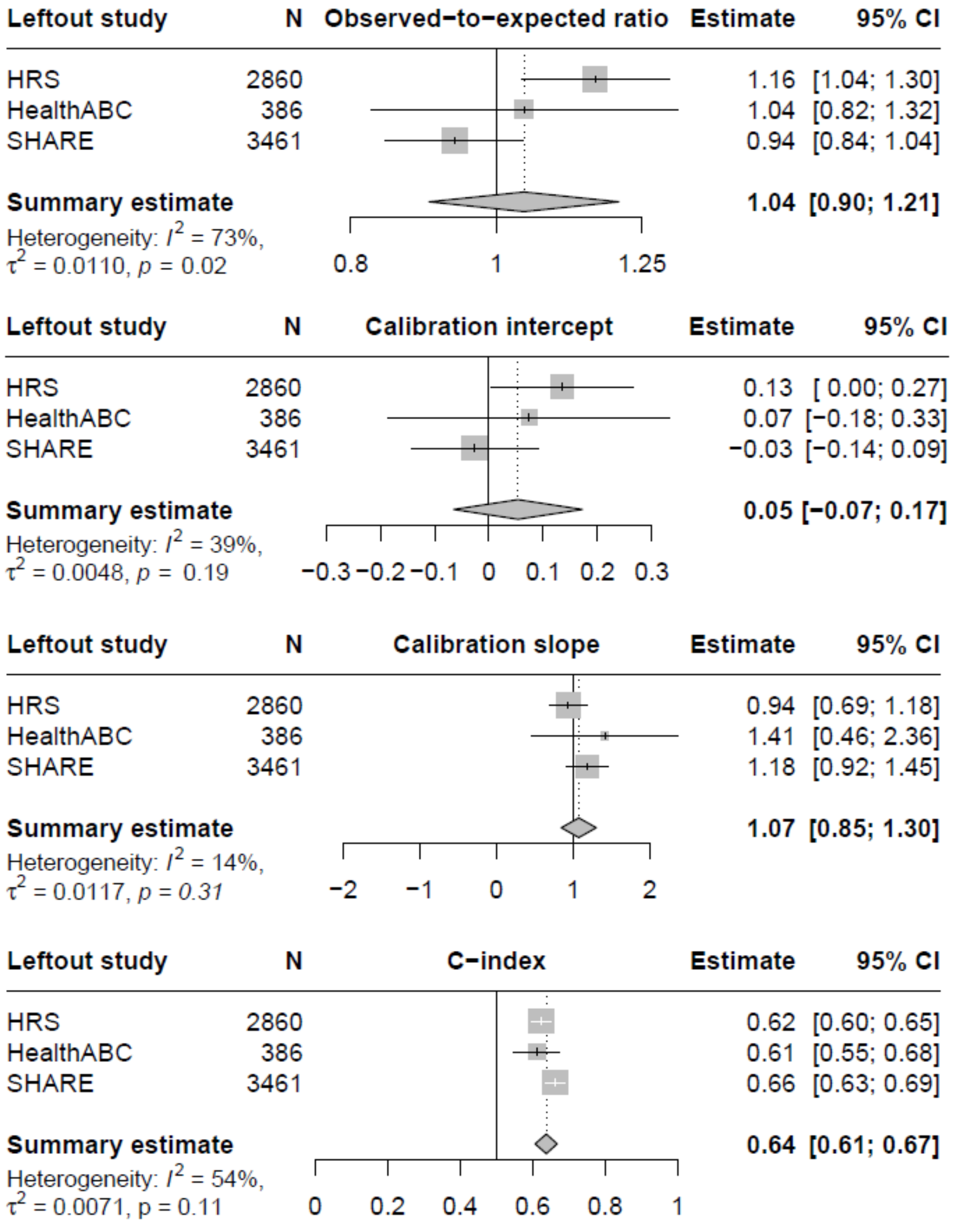

Performance measures not estimated for CoLaus due to only 6 participants still at risk by 5 years.

Supplementary Figure 4 Calibration plots from internal-external cross-validation

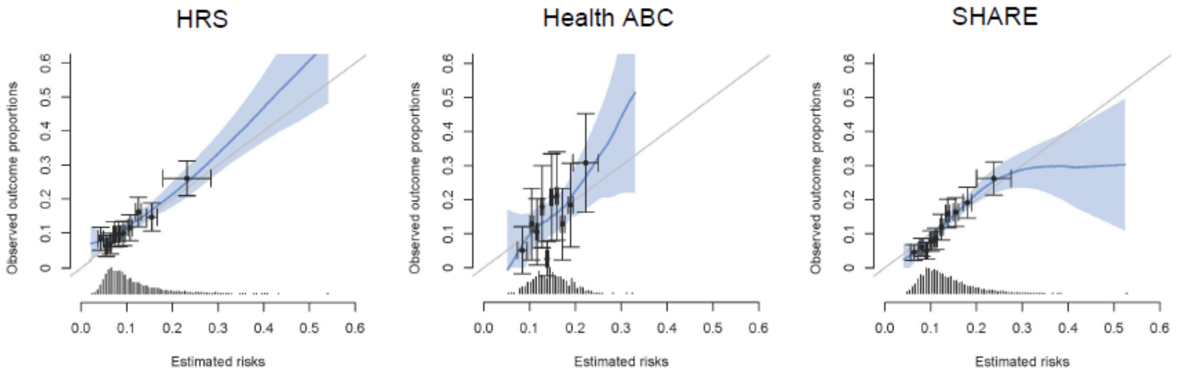

Performance measures not estimated for CoLaus due to only 6 participants still at risk by 5 years.

Supplementary Figure 5 Decision curve analysis

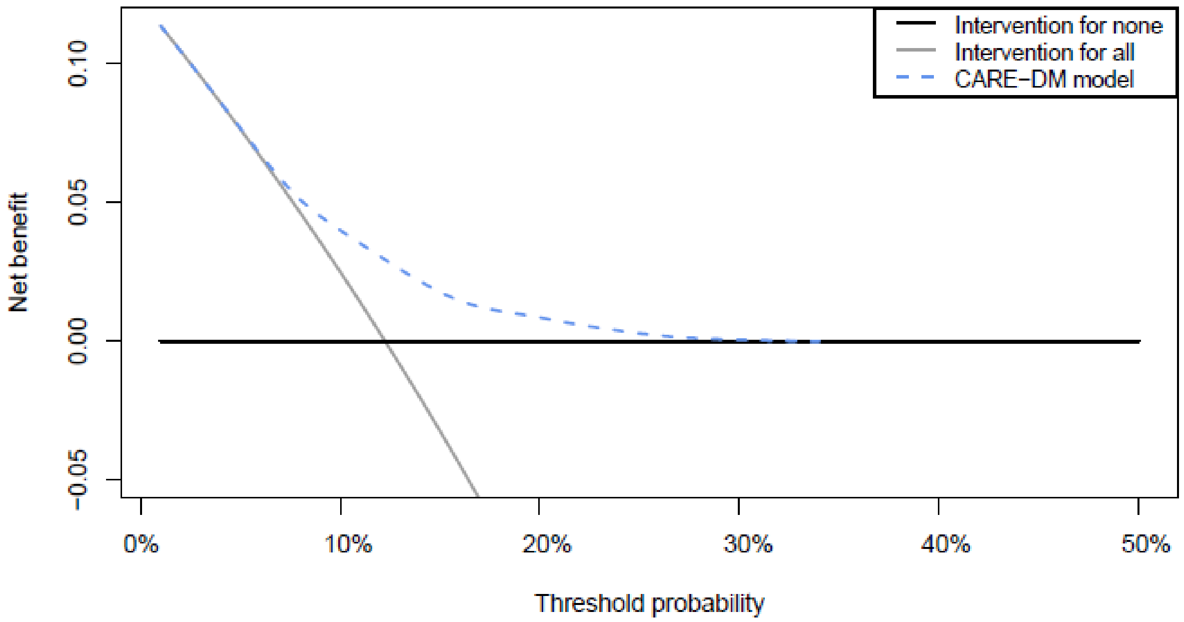

The decision curve indicates the 5-year net benefit (on the y-axis) of three clinical strategies (intervention for none, intervention for all or using the CARE-DM model) on the target population over a range of possible threshold probabilities (on the x-axis). Threshold probabilities reflect the decision-makers preference over sensitivity (low threshold probability) versus specificity (high threshold probability). The curve shows that the CARE-DM model would be the preferred strategy for threshold probabilities between 8% and 28%. Clinical guidelines from Australia and New Zealand consider a threshold probability of 10% or 15% for the 'high-risk' category of 5-year CVD risk.[36,37] The European Society of Cardiology considers a 10-year risk of 20% as 'very high risk', corresponding approximately to a threshold probability at 5-years of 10%.[38]

REFERENCES

1. Health and Retirement Study. (Cross-Wave Tracker File) public use dataset. Produced and distributed by the University of Michigan with funding from the National Institute on Aging (grant number NIA U01AG009740). Ann Arbor, MI; 2024.
2. Health and Retirement Study. (2006 HRS Core) public use dataset. Produced and distributed by the University of Michigan with funding from the National Institute on Aging (grant number NIA U01AG009740). Ann Arbor, MI.; 2021.
3. Health and Retirement Study. (2008 HRS Core) public use dataset. Produced and distributed by the University of Michigan with funding from the National Institute on Aging (grant number NIA U01AG009740). Ann Arbor, MI.; 2014.
4. Health and Retirement Study. (2010 HRS Core) public use dataset. Produced and distributed by the University of Michigan with funding from the National Institute on Aging (grant number NIA U01AG009740). Ann Arbor, MI.; 2021.
5. Health and Retirement Study. (2012 HRS Core) public use dataset. Produced and distributed by the University of Michigan with funding from the National Institute on Aging (grant number NIA U01AG009740). Ann Arbor, MI.; 2020.
6. Health and Retirement Study. (2014 HRS Core) public use dataset. Produced and distributed by the University of Michigan with funding from the National Institute on Aging (grant number NIA U01AG009740). Ann Arbor, MI.; 2017.
7. Health and Retirement Study. (2016 HRS Core) public use dataset. Produced and distributed by the University of Michigan with funding from the National Institute on Aging (grant number NIA U01AG009740). Ann Arbor, MI.; 2019.
8. Health and Retirement Study. (2018 HRS Core) public use dataset. Produced and distributed by the University of Michigan with funding from the National Institute on Aging (grant number NIA U01AG009740). Ann Arbor, MI.; 2022.
9. Health and Retirement Study. (2020 HRS Core) public use dataset. Produced and distributed by the University of Michigan with funding from the National Institute on Aging (grant number NIA U01AG009740). Ann Arbor, MI.; 2023.
10. Health and Retirement Study. (2006 HRS Exit) public use dataset. Produced and distributed by the University of Michigan with funding from the National Institute on Aging (grant number NIA U01AG009740). Ann Arbor, MI.; 2008.
11. Health and Retirement Study. (2008 HRS Exit) public use dataset. Produced and distributed by the University of Michigan with funding from the National Institute on Aging (grant number NIA U01AG009740). Ann Arbor, MI.; 2010.
12. Health and Retirement Study. (2010 HRS Exit) public use dataset. Produced and distributed by the University of Michigan with funding from the National Institute on Aging (grant number NIA U01AG009740). Ann Arbor, MI.; 2012.
13. Health and Retirement Study. (2012 HRS Exit) public use dataset. Produced and distributed by the University of Michigan with funding from the National Institute on Aging (grant number NIA U01AG009740). Ann Arbor, MI.; 2015.
14. Health and Retirement Study. (2014 HRS Exit) public use dataset. Produced and distributed by the University of Michigan with funding from the National Institute on Aging (grant number NIA U01AG009740). Ann Arbor, MI.; 2016.
15. Health and Retirement Study. (2016 HRS Exit) public use dataset. Produced and distributed by the University of Michigan with funding from the National Institute on Aging (grant number NIA U01AG009740). Ann Arbor, MI.; 2018.
16. Health and Retirement Study. (2018 HRS Exit) public use dataset. Produced and distributed by the University of Michigan with funding from the National Institute on Aging (grant number NIA U01AG009740). Ann Arbor, MI.; 2022.

17. Health and Retirement Study. (2020 HRS Exit) public use dataset. Produced and distributed by the University of Michigan with funding from the National Institute on Aging (grant number NIA U01AG009740). Ann Arbor, MI; 2023.
18. Health and Retirement Study. (2006 Biomarker Data) public use dataset. Produced and distributed by the University of Michigan with funding from the National Institute on Aging (grant number NIA U01AG009740). Ann Arbor, MI.; 2013.
19. Health and Retirement Study. (2008 Biomarker Data) public use dataset. Produced and distributed by the University of Michigan with funding from the National Institute on Aging (grant number NIA U01AG009740). Ann Arbor, MI.; 2013.
20. Health and Retirement Study. (2010 Biomarker Data) public use dataset. Produced and distributed by the University of Michigan with funding from the National Institute on Aging (grant number NIA U01AG009740). Ann Arbor, MI.; 2015.
21. Health and Retirement Study. (2012 Biomarker Data) public use dataset. Produced and distributed by the University of Michigan with funding from the National Institute on Aging (grant number NIA U01AG009740). Ann Arbor, MI.; 2015.
22. Health and Retirement Study. (2014 Biomarker Data) public use dataset. Produced and distributed by the University of Michigan with funding from the National Institute on Aging (grant number NIA U01AG009740). Ann Arbor, MI.; 2017.
23. Health and Retirement Study. (2016 Biomarker Data) public use dataset. Produced and distributed by the University of Michigan with funding from the National Institute on Aging (grant number NIA U01AG009740). Ann Arbor, MI.; 2020.
24. Health and Retirement Study. (2016 Venous Blood Study (VBS)) public use dataset. Produced and distributed by the University of Michigan with funding from the National Institute on Aging (grant number NIA U01AG009740). Ann Arbor, MI.; 2017.
25. SHARE-ERIC. Survey of Health, Ageing and Retirement in Europe (SHARE) Wave 6. Release version: 9.0.0. SHARE-ERIC; 2024. Data set. DOI:10.6103/SHARE.w6.900
26. SHARE-ERIC. Survey of Health, Ageing and Retirement in Europe (SHARE) Wave 7. Release version: 9.0.0. SHARE-ERIC; 2024. Data set. DOI:10.6103/SHARE.w7.900
27. SHARE-ERIC. Survey of Health, Ageing and Retirement in Europe (SHARE) Wave 8. Release version: 9.0.0. SHARE-ERIC; 2024. Data set. DOI:10.6103/SHARE.w8.900
28. SHARE-ERIC. Survey of Health, Ageing and Retirement in Europe (SHARE) Wave 8. COVID-19 Survey 1. Release version: 9.0.0. SHARE-ERIC; 2024. Data set. DOI:10.6103/SHARE.w8ca.900
29. SHARE-ERIC. Survey of Health, Ageing and Retirement in Europe (SHARE) Wave 9. Release version: 9.0.0. SHARE-ERIC; 2024. Data set. DOI:10.6103/SHARE.w9.900
30. SHARE-ERIC. Survey of Health, Ageing and Retirement in Europe (SHARE) Wave 9. COVID-19 Survey 2. Release version: 9.0.0. SHARE-ERIC; 2024. Data set. DOI:10.6103/SHARE.w9ca.900
31. SHARE-ERIC. Survey of Health, Ageing and Retirement in Europe (SHARE) Wave 6. SHARE Dried Blood Spots. Release version: 1.0.0. SHARE-ERIC; 2024. Data set. DOI:10.6103/SHARE.w6.DBS.100
32. Liu K, Marques-Vidal P. Sleep well, but be active. Effect of sleep and sedentariness on incidence of diabetes. *Prim Care Diabetes* 2023;**17**:454-459. doi: 10.1016/j.pcd.2023.08.002
33. SCORE2-Diabetes Working Group and the ESC Cardiovascular Risk Collaboration. SCORE2-Diabetes: 10-year cardiovascular risk estimation in type 2 diabetes in Europe. *European Heart Journal* 2023;**44**:2544-2556. doi: 10.1093/eurheartj/ehad260
34. Score Op working group, E. S. C. Cardiovascular risk collaboration. SCORE2-OP risk prediction algorithms: estimating incident cardiovascular event risk in older persons in four geographical risk regions. *European Heart Journal* 2021;**42**:2455-2467. doi: 10.1093/eurheartj/ehab312

35. Jackson C. flexsurv: A Platform for Parametric Survival Modeling in R. *Journal of Statistical Software* 2016;**70**:1 - 33. doi: 10.18637/jss.v070.i08
36. Nelson MR, Banks E, Brown A*, et al.* 2023 Australian guideline for assessing and managing cardiovascular disease risk. *Med J Aust* 2024;**220**:482-490. doi: 10.5694/mja2.52280
37. Ministry of Health. Cardiovascular Disease Risk Assessment and Management for Primary Care. In. Wellington; 2018.
38. Marx N, Federici M, Schütt K*, et al.* 2023 ESC Guidelines for the management of cardiovascular disease in patients with diabetes: Developed by the task force on the management of cardiovascular disease in patients with diabetes of the European Society of Cardiology (ESC). *European Heart Journal* 2023;**44**:4043-4140. doi: 10.1093/eurheartj/ehad192
