# Supplementary material for: Development and validation of the CARE-DM model to predict the cardiovascular risk in older persons with type 2 diabetes
Source: Eur J Prev Cardiol. 2025 May 14;33(1):44–52. doi: 10.1093/eurjpc/zwaf296 (PMC12771341; doi:10.1093/eurjpc/zwaf296)
Supplement: zwaf296_Supplementary_Data [file zwaf296_supplementary_data.zip › CVD prediction model_Supplement2_EJPC.pdf]

SUPPLEMENTARY APPENDIX 2

Supplement to: "Development and validation of the CARE-DM model to predict the cardiovascular risk in older persons with type 2 diabetes".

Instructions on how to calculate the CVD risk using CARE-DM:

Step 1

Calculate model variables (x1 to x14) as described in the table below.

**Risk factor**	**Model variable**
Age (in years)	x1 = Age - 70
Gender (woman versus man)	x2 = 1 if woman<br>x2 = 0 if man
Current smoking (yes vs no)	x3 = 1 if current smoker<br>x3 = 0 if no current smoker
Number of alcoholic drinks (< 1 per week vs 1 to 7 per week vs > 7 per week)	x4 = 1 if 1 to 7 alcoholic drinks per week<br>x4 = 0 if fewer than 1 alcoholic drink per week<br>x4 = 0 if more than 7 alcoholic drinks per week<br><br>x5 = 1 if more than 7 alcoholic drinks per week<br>x5 = 0 if fewer than 7 alcoholic drinks per week
BMI (in kg/m2)	x6 = BMI - 30
Use of antihypertensive medication (yes vs no)	x7 = 1 if using antihypertensive medication<br>x7 = 0 if not using antihypertensive medication
Use of cholesterol-lowering medication (yes vs no)	x8 = 1 if using cholesterol-lowering medication<br>x8 = 0 if not using cholesterol-lowering medication
Total cholesterol (in mmol/L)	x9 = Total cholesterol - 5
HDL cholesterol (in mmol/L)	x10 = HDL cholesterol – 1.3
Diabetes duration (≤ 5 years vs 5 to 10 years vs > 10 years)	x11 = 1 if diabetes duration over 5 to up to 10 years<br>x11 = 0 if diabetes duration 5 years or under<br>x11 = 0 if diabetes duration over 10 years<br><br>x12 = 1 if diabetes duration over 10 years<br>x12 = 0 if diabetes duration 10 years or under
HbA1c (in %)	x13 = HbA1c – 6.5
Use of diabetes medication (insulin or oral medication) (yes vs no)	x14 = 1 if using diabetes medication<br>x14 = 0 if not using diabetes medication

Step 2

Calculate the linear predictor by multiplying the model variables with their corresponding β-coefficients (log sub-hazard ratios) and summing all resulting values:

$$
\begin{aligned}
Linear\ predictor &= \sum Model\ variable\ \times\ \beta\ coefficient \\
&= x1 \times 0.054 + x2 \times -0.337 + x3 \times 0.318 + x4 \times -0.246 + x5 \times -0.116 \\
&+ x6 \times 0.019 + x7 \times 0.268 + x8 \times -0.123 + x9 \times 0.006 + x10 \times -0.006 \\
&+ x11 \times 0.038 + x12 \times 0.221 + x13 \times 0.080 + x14 \times -0.039
\end{aligned}
$$

**Risk factor**	**Model variable**	**β-coefficients (log sub-hazard ratios)**
Age (years)	x1	0.054
Gender	x2	-0.337
Current smoking	x3	0.318
Drinking 1 to 7 alcoholic drinks per week	x4	-0.246
Drinking > 7 alcoholic drinks per week	x5	-0.116
BMI	x6	0.019
Use of antihypertensive medication	x7	0.268
Use of cholesterol-lowering medication	x8	-0.123
Total cholesterol (in mmol/L)	x9	0.006
HDL cholesterol (in mmol/L)	x10	-0.066
Diabetes duration 5 to 10 years	x11	0.038
Diabetes duration > 10 years	x12	0.221
HbA1c (in %)	x13	0.080
Use of diabetes medication	x14	-0.039

Step 3

Calculate the shape and scale parameters of the Weibull distribution using the linear predictor.

**Parameter**	**Value**
Shape	1.065
Scale	$EXP(-\frac{(-6.651\ +\ linear\ predictor)}{1.065})$

Step 4

Calculate the risk of cardiovascular disease at a specific time (in months) using the shape and scale parameters.

$$
\begin{aligned}
Risk &= 1 - EXP(-((\frac{time\ in\ months}{scale})^{shape})) \\
&= 1 - EXP(-((\frac{time\ in\ months}{scale})^{1.065}))
\end{aligned}
$$

Example calculation of the 5-year risk of CVD for a 75-year-old woman:

**Risk factor**	**Value**	**Model variable**	**β-coefficient**	**Model variable * β-coefficient**
Age (in years)	75	x1 = 75 – 70 = 5	0.054	5 * 0.054 = 0.270
Gender (woman versus man)	Woman	x2 = 1	-0.337	1 * -0.337 = -0.337
Current smoking (yes vs no)	No	x3 = 0	0.318	0 * 0.318 = 0
Number of alcoholic drinks (<1 per week vs 1 to 7 per week vs >7 per week)	1 to 7 per week	x4 = 1	-0.246	1 * -0.246 = -0.246
		x5 = 0	-0.116	0 * -0.116 = 0
BMI	28	x6 = 28 – 30 = -2	0.019	-2 * 0.019 = -0.038
Use of antihypertensive medication (yes vs no)	Yes	x7 = 1	0.268	1 * 0.268 = 0.268
Use of cholesterol-lowering medication (yes vs no)	No	x8 = 0	-0.123	0 * -0.123 = 0
Total cholesterol (in mmol/L)	5	x9 = 5 - 5 = 0	0.006	0 * 0.006 = 0
HDL cholesterol (in mmol/L)	1.6	x10 = 1.6 – 1.3 = 0.3	-0.066	0.3 * -0.066 = -0.0198
Diabetes duration (≤5 years vs 5 to 10 years vs >10 years)	>10 years	x11 = 0	0.038	0 * 0.038 = 0
		x12 = 1	0.221	1 * 0.221 = 0.221
HbA1c (in %)	7	x13 = 7 – 6.5 = 0.5	0.08	0.5 * 0.080 = 0.040
Use of diabetes medication (yes vs no)	Yes	x14 = 1	-0.039	1 * -0.039 = -0.039
**Sum (linear predictor)**				**0.1192**

Shape = 1.065

Scale = EXP(- (-6.651 + 0.1192) / 1.065) = 460.8836

5-year risk = 1 - EXP(- ((60 / 460.8836) ^ 1.065)) = 0.108 = 10.8%

10-year risk = 1- EXP(- ((120 / 460.8836) ^ 1.065)) = 0.212 = 21.2%
